# Genome-wide analysis of fitness-factors in uropathogenic *Escherichia coli* during growth in laboratory media and during urinary tract infections

**DOI:** 10.1099/mgen.0.000719

**Published:** 2021-12-20

**Authors:** Vanesa García, Rasmus B. Grønnemose, Sergi Torres-Puig, Egle Kudirkiene, Mateo Piantelli, Shahana Ahmed, Thomas E. Andersen, Jakob Møller-Jensen, John E. Olsen, Ana Herrero-Fresno

**Affiliations:** ^1^​ Department of Veterinary and Animal Sciences, Faculty of Health and Medical Sciences, University of Copenhagen, Frederiksberg, Denmark; ^2^​ Laboratorio de Referencia de Escherichia coli (LREC), Departamento de Microbioloxía e Parasitoloxía, Facultade de Veterinaria, Universidade de Santiago de Compostela (USC), Lugo, Spain; ^3^​ Research Unit of Clinical Microbiology, University of Southern Denmark and Odense University Hospital, Odense, Denmark; ^4^​ Institute for Biochemistry and Molecular Biology, University of Southern Denmark, Odense, Denmark; ^5^​ Department of Developmental, Molecular & Chemical Biology, Tufts University School of Medicine, Boston, MA, USA

**Keywords:** essentiality, EZ-MOPS, fitness, human bacteriuria, LB, mouse cystitis, TraDIS, Uropathogenic *E. coli*, urinary tract infection

## Abstract

Uropathogenic *Escherichia coli* (UPEC) UTI89 is a well-characterized strain, which has mainly been used to study UPEC virulence during urinary tract infection (UTI). However, little is known on UTI89 key fitness-factors during growth in lab media and during UTI. Here, we used a transposon-insertion-sequencing approach (TraDIS) to reveal the UTI89 essential-genes for *in vitro* growth and fitness-gene-sets for growth in Luria broth (LB) and EZ-MOPS medium without glucose, as well as for human bacteriuria and mouse cystitis. A total of 293 essential genes for growth were identified and the set of fitness-genes was shown to differ depending on the growth media. A modified, previously validated UTI murine model, with administration of glucose prior to infection was applied. Selected fitness-genes for growth in urine and mouse-bladder colonization were validated using deletion-mutants. Novel fitness-genes, such as *tusA, corA* and *rfaG;* involved in sulphur-acquisition, magnesium-uptake, and LPS-biosynthesis, were proved to be important during UTI. Moreover, *rfaG* was confirmed as relevant in both niches, and therefore it may represent a target for novel UTI-treatment/prevention strategies.

## Data Summary

All raw data generated for this study have been submitted to the European Nucleotide Archive (ENA; https://www.ebi.ac.uk/ena/) under the study accession number PRJEB41961.

**Impact Statement**

Uropathogenic *E. coli* (UPEC) are the primary cause of urinary tract infections (UTIs) in humans. In order to develop novel approaches to prevent and control the disease, it is essential to understand which genes contribute to fitness of UPEC during UTI. In this work, the fitness-gene-sets in the UPEC UTI89 strain, during growth in lab media, human urine and during UTI in an improved mouse model were analysed and compared using transposon-directed insertion-sequencing (TraDIS). This study provides novel insight into the molecular mechanisms that UPEC employ to survive and/or grow in different environments, allowing us to gain knowledge about the biology of UPEC during infection and how this differs from life in the laboratory media. Several factors, such as some involved in LPS biosynthesis and others of new description, confirmed to be relevant during UTI, might be targeted by new therapeutics and/or prophylactics against UTI. The mouse model of UTI employed, where mice were administered glucose prior to infection, yielding decrease of urine concentration, and therefore mimicking conditions in humans, was shown to be useful for identification of fitness-gene-sets when analysing high-saturated transposon libraries.

## Introduction

Uropathogenic *E. coli* (UPEC) are responsible for >90% of cases of urinary tract infection (UTI) in humans. Based on the site of infection and severity, UTIs are classified into bacteriuria (bacteria in the urine), cystitis (inflammation of the bladder), pyelonephritis (inflammation of the kidney) and urosepsis (spread of bacteria to the blood) [1–3]. UTIs are the second most common infectious diseases in humans, accounting for ~150 million cases annually worldwide, and thus, represent an important problem in the health care systems and a cause of significant economic costs [4].

UPEC reside in the intestine, from where they contaminate the genitourinary system. Typically, UPEC strains express several virulence factors, including determinants for adhesion and persistence in the urinary tract [1, 5, 6]. Factors related to metabolic flexibility also play an important role in UTIs, since UPEC have to be able to grow in both the intestine and the urinary tract [3]. UPEC constitute a broad group of bacteria, and genes involved in UTIs may vary depending on the strain. In addition, host-related factors likely influence the set of UPEC genes that are involved in the infection, as revealed by transcriptomics and proteomics approaches [7–11].

In recent years, there has been an alarming increase in antimicrobial resistance among UPEC strains towards many of the relevant drugs, including those defined by the World Health Organization (WHO) as critically important for human health. Thus, there is an urgent need to find novel targets for antimicrobials to treat UTIs and to identify vaccine targets for prevention [3, 4]. In order to do so, it is essential to understand which bacterial genes are required for UPEC to colonize, grow and survive in the urinary tract during a UTI.

UTI89, belonging to multilocus sequence type 95 (ST95) is considered a prototype UPEC isolate, and it has been commonly used in studies of virulence genes associated to UTI [12, 13], however, a general characterization of fitness-genes for growth in laboratory media and human urine and during infection of mouse bladders in connection with UTI is still missing. Transposon-directed insertion-site sequencing (TraDIS) is a high-throughput technique, which combines traditional random transposon (Tn) mutagenesis with short-fragment DNA sequencing. It allows assaying large libraries of isogenic Tn-mutants in order to provide insights into gene essentiality, degree of fitness associated to each gene, gene-functions and genetic interactions [14, 15]. A Tn-insertion library is generated in a strain of interest with insertion of transposons into all non-essential-genes for the growth condition used to construct the library (input library). This library can then be screened under a specific test condition, for example during growth in different media and during progression of UTI (output library), and fitness related genes (i.e. genes important or required for growth/survival under the condition tested) can be identified by comparing the two libraries. Based on the number of sequence reads generated at the different Tn-insertion sites, the relative proportion of each mutant in the input and the output libraries can be estimated and the contribution of each gene to fitness under the tested condition can be quantitatively determined [16, 17]. In this work, we performed TraDIS to define the essential-genes required for growth of UPEC UTI89 *in vitro* (i.e. during growth in LB agar plates supplemented with kanamycin – Kn). Also, the fitness landscape of UTI89 during growth in frequently used lab media and human urine as well as during mouse cystitis was investigated.

**Fig. 1.**
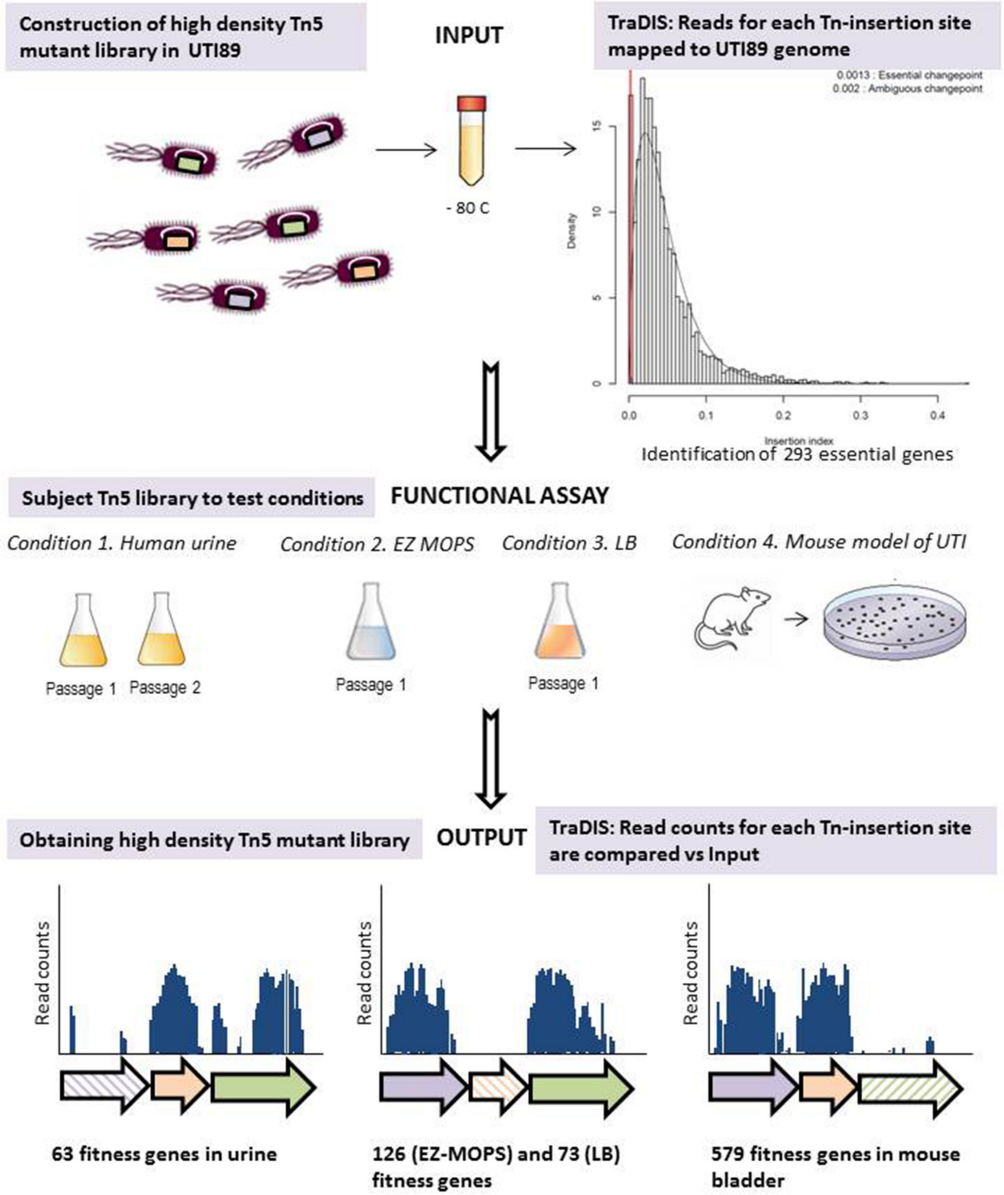
TraDIS as a research approach in UPEC to identify genes relevant for UTI and growth in laboratory media. Schematic representation of the UPEC UTI89 Tn5 mutant library and growth conditions tested in this work. The graph for identification of growth essential-genes has been obtained using the Bio::Tradis analysis pipeline (https://github.com/sanger-pathogens/bio-tradis).

The traditional mouse model of ascending UTI does not allow for testing of saturated Tn-mutant libraries due to a bottleneck effect during infection, partly caused by highly concentrated mouse urine. Therefore, here, we applied a modified model of UTI using slightly diuretic mice [18, 19] allowing the assessment of gene-fitness contribution when using complex Tn-libraries and under conditions that better reflect the human bladder environment. The experimental workflow is summarized in Fig. 1.

## Methods

### Bacterial strains, growth conditions and genome comparison

The strains used in the present work are listed in Table 1. *E. coli* UTI89 was originally isolated from a patient with an acute bladder infection [12] and its annotated genome of 5.18 Mb is publicly available [12, 13].

**Table 1.** Strains used in the present study

Strain	Relevant features	Source	Niche where a gene role was confirmed*
UTI89	*E .coli* wild-type, clinical isolate from cystitis patient	[12]	
UTI89-pKD46	UTI89 harbouring the plasmid pKD46 with λ red recombinase expressed from an arabinose inducible promoter (Gen^R^)	[22]	
UTI89 Rif^R^	*E .coli* UTI89 (Rif^R^)	This work	
*∆nhaA* ^1^	* E. coli* UTI89 lacking *nhaA* (Chl^R^)	This work	
*∆ybeY* ^1^	* E. coli* UTI89 lacking *ybeY* (Chl^R^)	This work	Urine, EZ-MOPS, LB
*∆tolQ* ^1^	* E. coli* UTI89 lacking *tolQ* (Chl^R^)	This work	
∆*tol* ^1^	* E. coli* UTI89 lacking the *tol* operon (Chl^R^)	This work	EZ-MOPS
*∆ompA* ^1^	* E. coli* UTI89 lacking *ompA* (Chl^R^)	This work	
*∆mgrB* ^1^	* E. coli* UTI89 lacking *mgrB* (Chl^R^)	This work	
*∆prc* ^1^	* E. coli* UTI89 lacking *prc* (Chl^R^)	This work	EZ-MOPS, LB
*∆UTI89_C1262* ^1^	* E. coli* UTI89 lacking *UTI89_C1262* (Chl^R^)	This work	Urine, EZ-MOPS, LB
*∆eda* ^1^	* E. coli* UTI89 lacking *eda* (Chl^R^)	This work	Urine
*∆rfaDC^1^*	* E. coli* UTI89 lacking *rfaD* and *rfaC* (Chl^R^)	This work	Urine, EZ-MOPS, LB
*∆rfaG^1,2^*	* E. coli* UTI89 lacking *rfaG* (Chl^R^)	This work	Urine, Mouse bladder
*∆relA* ^1^	* E. coli* UTI89 lacking *relA* (Chl^R^)	This work	Urine
*∆recB* ^1^	* E. coli* UTI89 lacking *recB* (Chl^R^)	This work	Urine, EZ-MOPS, LB
*∆tusA* ^1^	* E. coli* UTI89 lacking *tusA* (Chl^R^)	This work	Urine, LB
*∆atpH* ^1^	* E. coli* UTI89 lacking *atpH* (Chl^R^)	This work	
*∆atpF* ^1,2^	* E. coli* UTI89 lacking *atpF* (Chl^R^)	This work	Mouse bladder
*∆wzxE* ^1,2^	* E. coli* UTI89 lacking *wzxE* (Chl^R^)	This work	Mouse bladder
*∆corA* ^1,2^	* E. coli* UTI89 lacking *corA* (Chl^R^)	This work	Mouse bladder
*∆glnA* ^1^	* E. coli* UTI89 lacking *glnA* (Chl^R^)	This work	Urine, EZ-MOPS, LB
*∆ftsE* ^2^	* E. coli* UTI89 lacking *ftsE* (Chl^R^)	This work	Mouse bladder
*∆ypdE* ^2^	* E. coli* UTI89 lacking *ypdE* (Chl^R^)	This work	Mouse bladder
*∆himD* ^2^	* E. coli* UTI89 lacking *himD* (Chl^R^)	This work	Mouse bladder
*∆cutA* ^2^	* E. coli* UTI89 lacking *cutA* (Chl^R^)	This work	Mouse bladder
*∆phnO* ^2^	* E. coli* UTI89 lacking *phnO* (Chl^R^)	This work	Mouse bladder
*∆tam* ^2^	* E. coli* UTI89 lacking *tam* (Chl^R^)	This work	Mouse bladder
*∆sufA* ^2^	* E. coli* UTI89 lacking *sufA* (Chl^R^)	This work	

*Only shown for deletion-mutants showing a significant growth defect compared to WT.

^1^Genes mutated in UTI89 for validation of fitness effect during growth in human urine, LB and EZ-MOPS.

^2^Genes mutated in UTI89 for validation of fitness effect during UTI in mouse.

chloramphenicol, rifampicin, and gentamicin resistant, respectively; Chl^R^, Rif^R^ and Gen^R^.

A spontaneous rifampicin-resistant mutant of *E. coli* UTI89 (UTI89 Rif^R^) was obtained as reported [20]. Strains were grown at 37 °C on solid Luria broth (LB) media, in liquid LB (Sigma) or EZ-MOPS medium without glucose (Teknova). Antibiotics (Sigma) were added when required at the following concentrations: gentamicin (Gen) 15 µg ml^−1^, kanamycin (Kn) 50 µg ml^−1^, chloramphenicol (Chl) 30 µg ml^−1^ or rifampicin (Rif) 50 µg ml^−1^.

### Construction of the Tn5 mutant library and validation

Competent cells of *E. coli* UTI89 were prepared as previously described with slight modifications [21, 22]. Briefly, overnight cultures of the isolate were 1 : 100 diluted in LB medium and grown to an OD=0.5–0.6, then harvested by centrifugation, and washed three times; with 1× volume of cold water, 0.5× of cold water and 0.05× volume of 10 % cold glycerol. Finally, they were re-suspended in a 0.002× volume of 10 % cold glycerol and kept on ice. Aliquots of 60 µl competent cells were stored at −80 °C until use. Then, 1 μl EZ-Tn5<KAN-2>Tnp Transposome (Epicentre) was electroporated into an aliquot of competent cells of the strain following the manufacturer´s instructions. After electroporation, cells were resuspended in 1 ml Super Optimal broth with Catabolites repression (SOC) medium, incubated at 37 °C for 2 h and then cultured overnight at 37 °C on large, 15 cm Ø LB agar plates supplemented with Kn. c.f.u. counts were performed by plating tenfold dilutions on LB agar plates containing Kn and incubated overnight at 37 °C. Kn-resistant colonies from each plate were collected into sterilized LB supplemented with glycerol 20 % (v/v) using a sterilized bacteriological spreader and transferred into 50 ml tubes. After thorough mixing, the tubes were stored at −80 °C. Each tube contained between 10 000 and 45 000 mutants. The final input library consisted of 1.65×10^5^ mutants, generated by pooling mutants from nine tubes. Then, 1 ml aliquots of each expanded library, containing approximately 1×10^10^ c.f.u each, were stored at −80 °C. The input library was assessed for random insertion of the transposon at multiple sites across the genome using the PCR-based protocol ‘random amplification of transposon ends’ (RATE) prior to sequencing as described [23].

### Growth assays in human urine, EZ-MOPS and LB

The experimental workflow (Fig. 1) consisted of the UTI89 input library screening in EZ-MOPS, LB, human urine and the mouse model of UTI. Freshly voided urine was collected from two healthy female individuals with no history of UTI or antibiotic use within the last 2 months. The urine was pooled, filter-sterilized and used right after filtration as described elsewhere [22]. Approximately 1×10^9^ viable Tn-mutants were used to inoculate 9.9 ml of filtered urine and then incubated at 37 °C for 20 h to generate the output library: ‘output UTI89_urineT20’. One additional passage was performed; 100 µl from passage 1 containing approximately 1×10^9^ cells were transferred into 9.9 ml of fresh urine and incubated for further 20 h at 37 °C to generate ‘output UTI89_urineT40’. Assays in EZ-MOPS without glucose and LB were performed the same way to generate ‘output UTI89_MOPST20’ and ‘output UTI89_LBT20’. All growth assays were performed in duplicate on different days in order to obtain biological replicates for further analysis (Table 2). The output libraries obtained were directly processed for DNA extraction and sequencing (see below).

**Table 2.** Parameters for TraDIS data-sets in the current study.

Library (condition)	Total reads^1^	Reads mapped (%)^2^	Total UIS	Total seq len/Total UIS
**Input**				
UTI89_1 (input 1)	11 574 067	10 372 421 (89.6)	190 809	27.1
UTI89_2 (input 2)	8 331 970	8 049 946 (96.6)	149 998	27.8
UTI89_1+UTI89_2 (combined)	19 906 037	18 422 370 (92.5)	222 483	23.3
**Output (urine**)				
UTI89_urineT20_1	7 552 213	7 267 934 (96.2)	156 442	33.1
UTI89_urineT20_2	12 016 059	11 426 136 (95.1)	170 470	30.4
UTI89_urineT40_1	9 835 979	9 456 427 (96.1)	170 137	30.4
UTI89_urineT40_2	14 345 388	13 820 140 (96.3)	107 583	48.1
**Output (EZ-MOPS**)				
UTI89_MOPST20_1	8 113 704	6 891 908 (84.9)	164 719	31.4
UTI89_MOPST20_2	6 051 946	5 323 289 (84.9)	153 221	33.8
				
**Output (LB**)				
UTI89_LBT20_1	10 073 550	9 319 007 (92.5)	163 924	31.6
UTI89_LBT20_2	8 420 136	7 946 561 (94.4)	159 500	32.5
**Output (mice**)				
UTI89_V_1 (single mouse_V)	7 157 630	6 873 996 (96.1)	67 213	77.1
UTI89_V_2	12 049 930	11 465 874 (95.1)	75 755	68.4
UTI89_V1+UTI89_V2 (V combined)	19 207 560	18 339 871 (95.5)	85 268	60.7
UTI89_W_1 (single mouse_W)	6 535 767	6 059 389 (92.7)	40 090	129.3
UTI89_W_2	7 192 774	6 846 954 (95.2)	42 668	121.4
UTI89_W1+UTI89_W2 (W combined)	13 728 541	12 906 342 (94.1)	50 876	101.8
UTI89_Z_1 (11 mice)	7 197 671	6 779 735 (94.2)	84 124	61.6
UTI89_Z_2	7 962 478	7 570 978 (95.1)	84 334	61.4
UTI89_Z1+UTI89_Z2 (Z combined)	15 160 149	14 350 712 (94.7)	95 732	54.2

^1^Number of sequence reads with matching online barcode.

^2^Number of mapped sequence reads against *E. coli* UTI89 genome (% of the raw data).

UIS, Unique Insertion Sites.

### Infection of the mouse model of UTI

The UTI89 Tn-library was screened in the mouse model of ascending UTI in order to investigate fitness-genes during early colonization of the bladder. Infection was carried out as described [22, 24] with some modifications. Mice (*N*=11) were given water supplemented with 5 % glucose for 16 h prior to infection, as previously reported, to reduce mouse urine concentration values (USG) to the levels of human urine [18, 19]. USG were estimated before and after treatment with glucose as described [25]. Approximately 4–5×10^7^ Tn-mutants resuspended in 50 µl of Dulbecco’s PBS (DPBS) (Sigma) were inoculated trans-urethraly into each of the 11 anesthetized 8–9-week-old C3H/HeN female mice using a sterile catheter. Mice were infected and left for 6 h before analysis. c.f.u. counts of tenfold dilutions of the inoculum were performed on LB-agar plates in order to quantify the inoculated bacteria.

At the end of the experiment, mice were euthanized by cervical dislocation. The bladders from individual mice were excised. Bladders from three mice were recovered in 5 ml sterile DPBS each, and bladders from the remaining eight mice were pooled as four and four bladders per pool in 5 ml DPBS. Samples (*N*=5; from three single mice and from four and four mice) were kept on ice and homogenized with a mechanical tissue homogenizer (IKA Ultra-Turrax T25 rod disperser). Tenfold dilutions were performed from 100 µl of each sample and plated on LB agar plates, which were incubated overnight at 37 °C to determine c.f.u. counts. The remaining 4.9 ml of bladder suspensions were plated onto 15 cm Ø agar plates supplemented with Kn (0.3–0.5 ml suspension per plate) and incubated overnight at 37 °C. Colonies were collected as described for the input library above and output libraries were obtained. Then, 1 ml from each of the generated output libraries were eventually collected in order to obtain one composite output library representing all the 11 mice. Next, 1 ml aliquots of each expanded library containing approximately 1×10^10^ c.f.u. were prepared and stored at −80 °C. Information on the samples and output libraries obtained from the mouse assays are shown in Table S1, available in the online version of this article.

### DNA extraction, quality control and quantification

Genomic DNA was purified using the DNeasy blood and tissue kit (Qiagen) from 500 μl (approximately 5×10^9^ cells) of the input and the output libraries from samples V, W and Z (mouse experiments) (Table S1), as well as from 4 ml of output libraries ‘UTI89_urineT20’, and ‘UTI89_urineT40’, 1 ml of ‘UTI89_MOPST20’ and 1 ml of ‘UTI89_LBT20’, containing an estimated 1×10^9^ c.f.u. each. Quality of DNA was confirmed by NanoDrop (Thermo Fisher). DNA samples with parameters 280/260 between 1.8 and 2.0 and 260/230 between 2.0 and 2.2, were processed for further sequencing. DNA concentration was estimated by using Qubit dsDNA HS Assay Kit (Thermo Fisher).

### Sequencing of input and output libraries: multiplexed TraDIS

Preparation of libraries for sequencing was performed as described [15] with some modifications. Briefly, approximately 2–3 µg of DNA resuspended in 130 µl sterilized H_2_O were sheared using Covaris M220 (Covaris) into approximately 300 bp fragments following the manufacturer's recommendations with some modifications (i.e. a treatment time of 110 s was applied). Size distribution of fragments, DNA quality and quantity were checked using the Agilent High Sensitivity DNA Kit in the Agilent 2100 Bioanalyzer System (Agilent Technologies) after fragmentation. The subsequent steps of DNA end repair, DNA end adenylation and adapter ligation (NEBNext End Repair Module, NEBNext dA-Tailing ModuleNEBNext and Quick Ligation Module; New England Biolabs) were carried out following the manufacturer’s recommendations for each kit, and the adapter splinkerette design strategy was performed as described [15]. Enrichment of adapter-ligated fragments (≥100 ng) containing Tn-insertion by PCR was performed using a Tn-specific P5 forward primer (100 µM) and a TraDIS-P7 primer (SpIAP5.x) (10 µM) [15, 26] (Table S2) and a 19 cycle PCR followed by qualitative and quantitative verification through Bionalyzer and qPCR. Ampure XP beads (Ramcon) purification was performed after each specific step as described [15]. DNA samples and primers (Table S2) were loaded on a MiSeq platform using a MiSeq reagent kit V2 (50 cycles) (Illumina) following the recipe previously described [15]. All primers used for TraDIS are listed in Table S2.

The TraDIS sequence data from this study were deposited on the European Nucleotide Archive (ENA) under the study accession number PRJEB41961.

### Sequence data analysis and statistics

Analysis of sequencing data was performed essentially as described [15] using the Bio::Tradis analysis pipeline (https://github.com/sanger-pathogens/Bio-Tradis) with some modifications.

First, the reads obtained after the MiSeq run were processed using a custom *fq2bam.pl* script, which takes each tag from the read name and adds it to the front of the read and then converts the file into SAM format. Samtools were used to convert a produced SAM file into BAM format, which was used for further analyses with *check_tradis_tags* and *add_tradis_tags* scripts, both part of Bio::Tradis pipeline.

The next step in the read analysis was performed using the *bacteria_tradis* mapping pipeline. Briefly, the FASTQ files obtained were filtered for ten bases matching the 3′ end of the transposon. These transposon tags were trimmed out from the resulting reads, and the trimmed reads were mapped to the UTI89 reference genome (acc. number NC_007946.1) using the SMALT short-read mapper (https://www.sanger.ac.uk/tool/smalt-0/). The accurate insertion site of the transposon across the genome was estimated and unique insertion sites (UISs) and read counts were determined per gene. Further analysis was carried out using R scripts included into the Bio::Tradis pipeline. The read counts and UISs were visualized using Artemis version 17.01 [27], and circular genome diagrams were obtained by DNAPlotter version 17.01 [28].

Gene essentiality was evaluated using the analysis script *tradis_essentiality.R*, which employs a statistical analysis establishing a bimodal distribution of insertion indexes (IIDs) (number of insertions per gene divided by gene length) for non-essential-genes (gamma) and essential-genes (exponential). Log_2_ likelihood ratios (LLR) were calculated between the fitted distributions, and a gene was classified as essential if showing a LLR < −2, leading to an essentiality cutoff at an IID of 0.0013 in our data. A gene was classified as nonessential if showing a LLR >2, giving an insertion index cutoff of 0.002. IIDs falling between these two values were classified as ‘ambiguous’.

To identify UPEC fitness-genes during human bacteriuria, mouse cystitis, growth in EZ-MOPS and growth in LB, the *tradis_comparisons.R* script, which employs bio-conductor package edgeR [29], was used to establish significant differences in read counts between input and output pools among non-essential-genes. The TMM (trimmed mean of M values) normalization was applied, and tagwise dispersion was estimated. *P* values were corrected for multiple testing by the Benjamini–Hochberg method, and genes with a corrected *P* value (*Q* value) of <0.001 and an absolute log_2_ fold change difference (logFC) of < −2 were considered significant. Genes deemed to be fitness-genes for growth in urine and for UTI in the mice model (only genes with a LogFC < −7 in composite sample UTI89_Z) were re-annotated using the Uniprot-database [30]. The cut-off criteria LogFC >2 and *Q*<0.001 would define the so-called ‘anti-fitness-genes’ (i.e. those expected to have a disadvantageous effect in the test condition). Essential-genes for growth on plates supplemented with Kn, which showed zero or few transposon insertions and fitness-genes were further functionally categorized using the EggNOG database [31, 32] that searches for Cluster of Orthologous Groups (COG). They were plotted as fractions of the total number of genes in each functional category in the UTI89 reference genome.

### Construction and quality control of individual deletion-mutants

Specific mutants of *E. coli* UTI89 (*N*=26, Table 1) were constructed using the λ-Red recombinase method as described [33]. The primers used are listed in Table S3. Deletion-mutants were verified by PCR (Table S3). Whole-genome sequencing of the mutant strains was carried out using the Illumina MiSeq (Illumina). Reads were assembled and contigs were aligned using the CLC Workbench Software (CLC Bio-Qiagen). Gene-specific deletion in each mutant was confirmed through comparative genomic analysis of the genome of the mutant strain versus the UTI89 wild-type genome sequence using the CLC software. The chloramphenicol resistance cassette was removed from five mutants; Δ*eda*, Δ*tusA*, Δ*cutA*, Δ*tam* and Δ*relA,* following the method described by Datsenko and Wanner [33].

### Validation of the TraDIS analysis and statistics

The wild-type (WT) *E. coli* UTI89 and the mutants were compared for the ability to grow in human urine, EZ-MOPS and LB and/or to infect the mouse model of UTI. For the growth assays, overnight cultures of WT and mutant strains were adjusted to an OD_600_ of 0.05 in a final volume of 10 ml and incubated with shaking (180 r.p.m.) at 37 °C. The OD_600_ was determined every hour from *t*=0 h to *t*=8 h, and at *t*=24 h post-inoculation. A growth curve for each strain was obtained, and growth comparison was determined visually. *E. coli* UTI89 Rif^R^ and selected mutants (Δ*corA,* Δ*nhaA,* Δ*relA,* Δ*wzxE,* Δ*prc,* Δ*ompA,* Δ*mgrB,* Δ*rfaDC,* Δ*atpF,* Δ*atpH,* Δ*rfaG* and Δ*tol*) were also co-cultured in filtered human urine to compare growth ability in competition experiments, as reported [22]. For these assays, overnight cultures of each strain were adjusted to an OD of 0.1, mixed and inoculated at a final concentration of approximately 1×10^7^ c.f.u. ml^−1^ (ratio 1 : 1) in 5 ml of urine as determined by performing c.f.u. counts of the inoculum. Suspensions were incubated at 37 °C with shaking for 24 h. Samples were collected every second hour from *t*=0 to *t*=8 h and then at *t*=20 h and *t*=24 h post-inoculation, and tenfold dilutions were plated on LB agar plates supplemented with Rif (for detection of UTI89 Rif^R^) or Chl (for detection of mutant strains), respectively. Plates were incubated overnight at 37 °C, c.f.u. counts were performed, and growth curves were generated. The experiments were performed at least in triplicates.

The potential cost of the generated Rif-mutation was tested by comparing the growth in competition of *E. coli* UTI89 and *E. coli* UTI89 Rif^R^ in LB, EZ-MOPS and urine as described above. Similarly, the cost of the Chl-resistance in Δ*eda*, Δ*tusA*, Δ*cutA*, Δ*tam* and Δ*relA* was assessed during growth in competition of the deletion mutant with and without the resistance cassette.

Competition infection experiments were performed to identify fitness-genes during UTI in the mouse model. A few colonies of WT UTI89 and mutant, directly collected from plates after overnight growth, were each re-suspended in 5 ml of DPBS. The OD was adjusted to 0.5 with DPBS, and equal volumes of the strains were mixed to obtain the inoculum. c.f.u. counts were performed in order to enumerate the inoculated bacteria. Groups of 5–6 8-week-old C3H/HeN female mice were administered 5 % glucose in drinking water for 16 h and then infected trans-urethraly with 50 µl of the 1 : 1 (WT:mutant) mixture as described above. Mice were sacrificed at 6 h post-infection by cervical dislocation, and the bladder was excised and recovered in 5 ml sterile DPBS. The bladders were kept on ice and homogenized with a mechanical tissue homogenizer. Tenfold dilutions were performed and plated on LB agar plates supplemented with Rif or Chl for detection of WT and mutants, respectively. Plates were incubated at 37 °C overnight. Competitive infection index (CI) was calculated as the mutant c.f.u./WT c.f.u. ratio found in the bladder divided by the mutant c.f.u./WT c.f.u. ratio of the inoculum multiplied by 100, as previously described [34].

### Statistical analysis

Statistical significance was determined by one-sample *t*-test or one-way ANOVA with Sidak’s post-test (for comparison of USG in the mice experiments and of ODs and Log10 c.f.u. ml^-1^ in the growth assays) or one-way ANOVA and Dunnett's multiple comparison test (for comparison of CIs in the mice competitive assays) using GraphPad Prism (version 8.3.0) (GraphPad Software Inc.). A *P-*value (*P*) below 0.05 was considered significant.

## Results

### Verification and analysis of the UTI89 input and output Tn-libraries

A Tn*5* mutant input library was generated in the *E. coli* strain UTI89, consisting of approximately 1.65×10^5^ mutants. Random Tn-insertions throughout the bacterial genome were verified by the PCR-based RATE protocol performed on randomly selected mutants (data not shown).

TraDIS was carried out on input and output libraries, with the latter ones consisting of UTI89 Tn-library grown in EZ-MOPS, LB, human urine, and the same library tested in a modified mouse model of UTI to identify fitness-genes of UTI89 for these conditions. The Tn-insertion sites and immediate adjacent DNA regions were sequenced in 16 libraries. Number of reads obtained for each library ranged between 6.05 to 14.3 million, and when reads from replicates were combined, it ranged from 13.7 to 19.9 million for each condition assayed (Table 2). Between 84.9 % and 96.6 % of reads were found to map accurately to the UTI89 reference genome (Table 2).

### Essential-genes in *E. coli* UTI89 and comparison with other *E. coli* strains

Single gene deletion libraries are considered the gold standard for identifying essential-genes, and TraDIS is frequently applied for this purpose [35]. In this work, we used our input library to identify the essential-genes in *E. coli* UTI89 defined as the set of genes required for growth on LB agar supplemented with Kn, as reported [21].

**Fig. 2.**
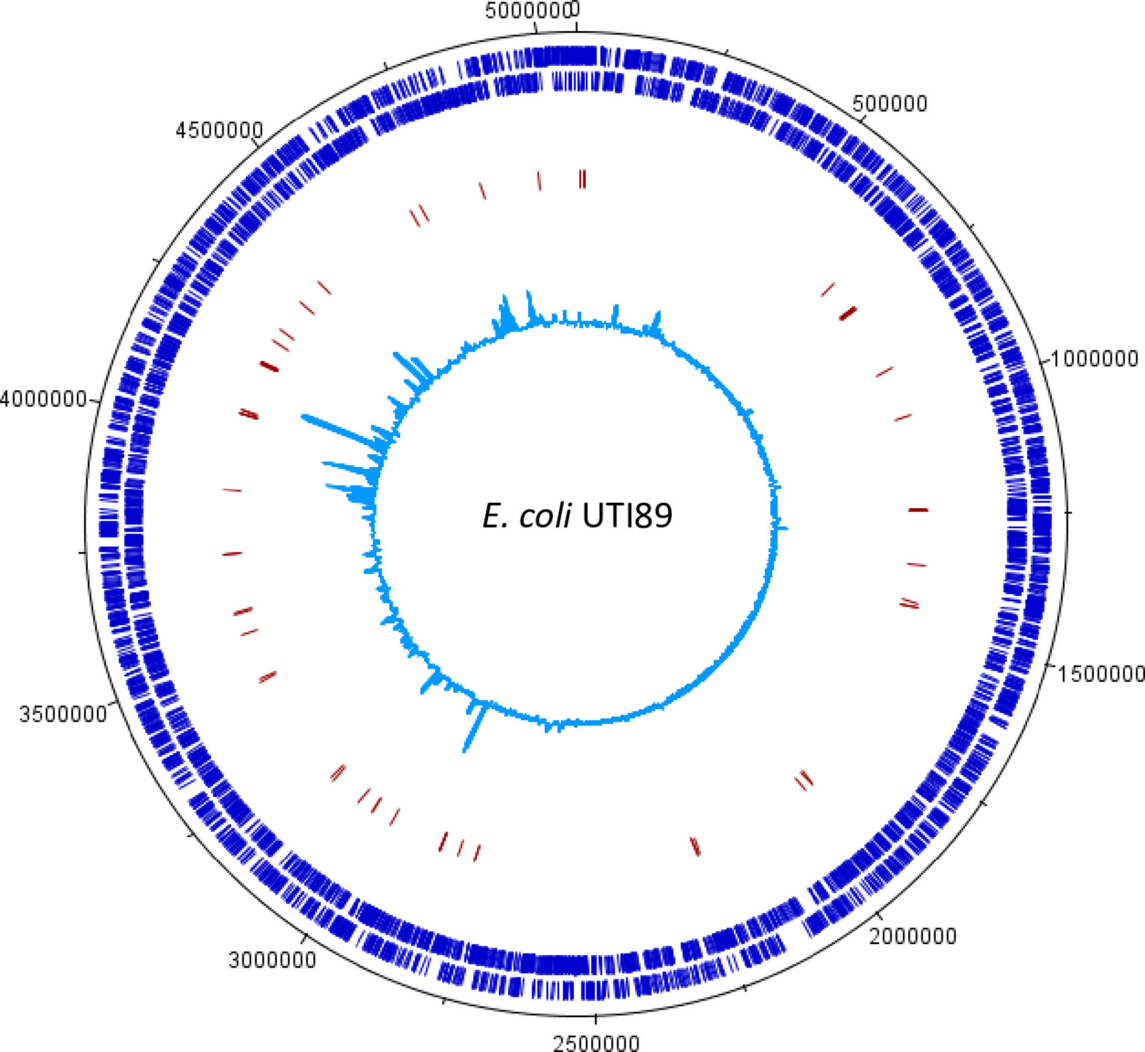
Genome-wide transposon insertion sites mapped to *E. coli* UTI89 strain. The outermost track in black marks the *E. coli* genome in base pairs starting at the annotation origin. The next inner track (dark blue) corresponds to sense and antisense CDS, respectively, followed by a red track depicting the fitness-genes predicted by TraDIS in UTI89 during growth in human urine. The innermost circle (light blue) corresponds to the frequency and location of transposon insertion sequences mapped successfully to the *E. coli* UTI89 genome after identification of a transposon sequence. This figure was created using DNAPlotter.

For analysis on gene essentiality, sequencing reads from duplicates of the input library were combined to maximize the coverage resulting in approximately 20 million transposon-tagged reads, of which approximately 18 million specifically mapped to the UTI89 chromosome, with 222 483 UISs, representing an average of one insertion site every 23.3 bps (Table 2). The resulting Tn*5* insertion profile across the genome of *E. coli* UTI89 is shown in Fig. 2. A total of 293 genes were categorized as essential (Fig. 1, Table S4).

Previous studies based on similar TraDIS approaches, and where essentiality was analysed under the same growth conditions as here, revealed that the essential-gene-lists for the reference strain *E. coli* K12 BW25113 and UPEC EC958 included 358 and 315 genes, respectively [21, 35]. Of the 293 essential-genes identified in UTI89 in this work, 198 (67.6 %) and 188 (64.2 %) were shared with K-12 BW25113 and UPEC EC958, respectively, and 66 genes (22.5%) were unique to UTI89. The uropathogen, EC958, had 231 (73.3 %) of the essential-genes in common with the *E. coli* K-12 reference strain [21].

**Fig. 3.**
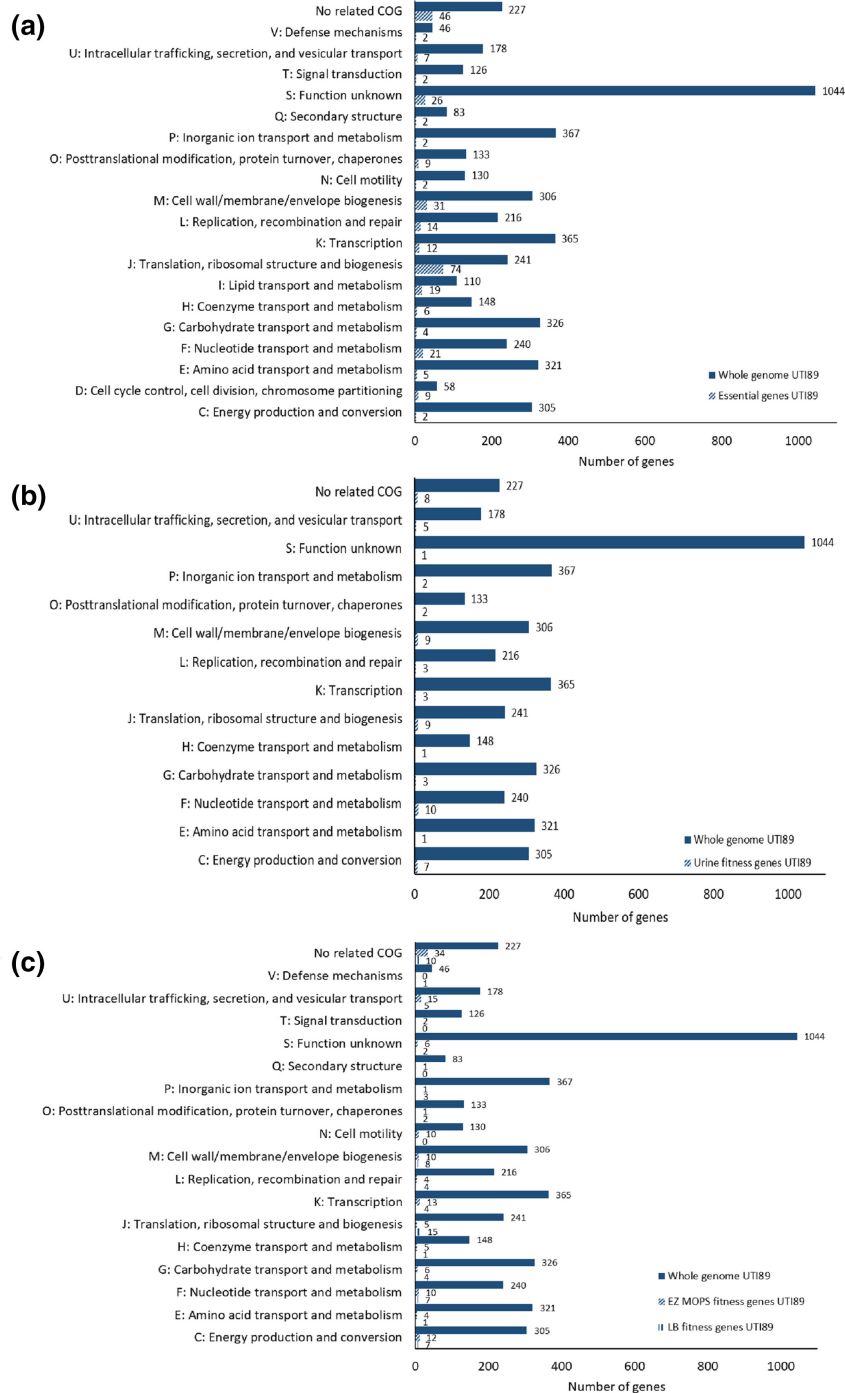
Functional classification of essential- and fitness-genes in UPEC UTI89. Essential-genes for growth in LB agar media supplemented with Kn (a) and fitness-genes for growth in human urine (b) predicted in *E. coli* UTI89 were functionally categorized using the EggNOG database (illustrated on the vertical axis). (c) shows the functional classification of fitness-genes predicted in *E. coli* UTI89 for growth in EZ-MOPS and LB media. The numbers indicate the essential/fitness-genes in each functional category compared with the total number of genes in the reference strain and belonging to the same category.

Protein-coding genes identified as essential for growth were assigned to a functional category using the COG database (Fig. 3). Four genes were categorized as belonging to two different COGs in UTI89. Genes involved in translation, ribosomal structure and biogenesis (COG J) represented 25.2 % of the total number of essential-genes. The second most frequent category included genes playing a role in cell wall/membrane/envelope biogenesis (10.6%) (COG M). Other frequent categories encompassed genes involved in nucleotide transport and metabolism (7.2 %) (COG F), lipid transport and metabolism (6.5 %) (COG I), replication, recombination and repair (4.8 %) (COG L) and transcription (4.1 %) (COG K). A relatively high proportion of the essential-genes; 24.6 %, encoded proteins of unknown function, or the genes were not categorized as belonging to any COG. The first and second most frequent categories in UPEC UTI89 were the same detected as the most abundant in the essential-gene list of UPEC EC958 [21]. In general, the same COGs groups were identified as essential in the two UPEC isolates, however, the number of protein-coding essential-genes varied in each category between the strains.

### Identification of UTI89 fitness-genes during growth in laboratory media

**Fig. 4.**
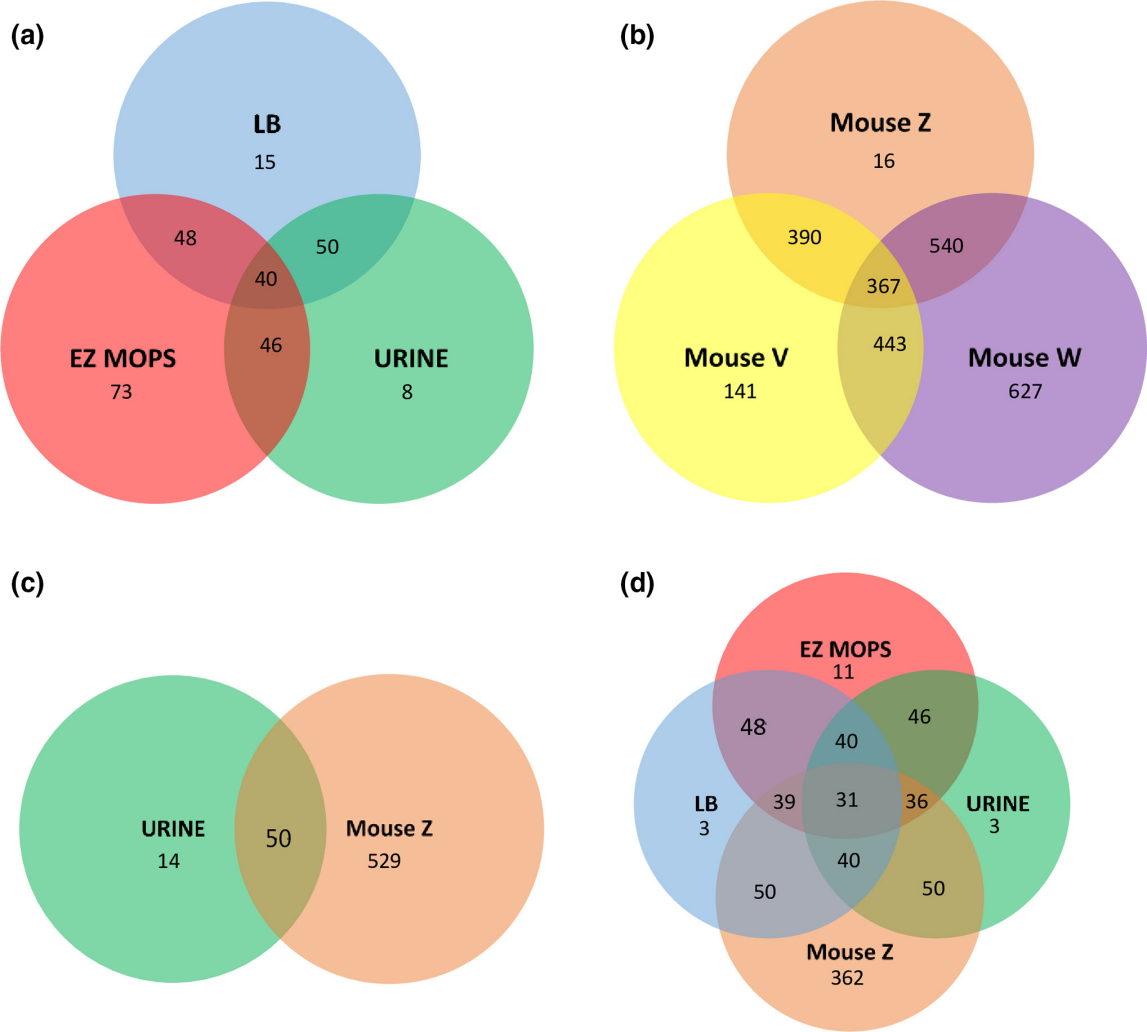
Venn diagrams showing the number of common and differential predicted fitness-genes in UPEC UTI89 between the tested conditions using TraDIS. (a) Fitness-genes during growth in LB, EZ-MOPS and human urine. (b) Fitness-genes during mice infection. (c) Fitness-genes during UTI caused by *E. coli* UTI89. (d) Fitness-genes during growth in LB, EZ-MOPS, human urine and mice infection. Mouse V: list of fitness-genes for single mouse V, mouse W: list of fitness-genes for single mouse W, mouse Z: list of fitness-genes for sample Z representing all 11 mice under study (see Table S6 for more details).

We applied TraDIS to identify fitness-genes in UTI89 during growth in defined laboratory media, LB and EZ-MOPS. LB is the most widely used nutritionally rich standard medium to grow *E. coli* and other Gram-negative bacteria, since it allows fast growth and high yields. However, LB composition is not well-defined and introduces high variability among batches [36, 37]. Here, we aimed to identify fitness-genes for growth of *E. coli* UTI89 in LB and in a defined rich medium, EZ-MOPS with no glucose added. A total of 73 and 126 fitness-genes were detected during growth in the media, 48 of which were shared between both growth conditions (Fig. 4a, Table S5). It should be noted that the higher number of fitness-genes in EZ-MOPS include possible essential-genes for growth in this media (i.e. if the input library had been generated in EZ-MOPS agar plates), a gene set, which is currently unknown. The shared genes were classified as associated to replication/transcription/translation/post-translation (*N*=12), hypothetical proteins (*N*=10), ATP synthesis (*N*=8), other metabolic functions (*N*=7), membrane biogenesis (*N*=6), tol-proteins (*N*=4) and others (*N*=1) (Table S5). Among the UTI89 genes affecting fitness in LB, 21 (28.8 %) were previously identified as fitness-genes in K-12 BW25113 cultured in LB (Table S5) in a study where fitness-genes were obtained via TraDIS [35]. The shared pool included genes involved in translation, ribosomal structure and biogenesis, transcription, post-translation modifications or replication (*N*=12), purine metabolism (*N*=1), other metabolic genes (*N*=6), and a gene encoding for a hypothetical protein (*N*=1).

### Identification of UTI89 fitness-genes during growth in human urine

A total of 63 genes were found to be important for growth in urine after 20 h of growth, and one additional gene was identified, when the resulting pool was subjected to further 20 h of growth (Table 3). Overall, 46 and 50 of the genes identified as fitness-genes in EZ-MOPS and LB, respectively, were also categorized as fitness-genes in UTI89 during growth in urine, and 40 were common to the pool of fitness-genes detected for the three conditions (Fig. 4a, Table S5). The shared pool encompassed genes related to replication, transcription, translation or encoding ribosomal functions (*N*=10), genes encoding hypothetical proteins (*N*=7), genes involved in ATP synthesis (*N*=7), tol-genes (*N*=4), genes encoding membrane proteins (*N*=5) and others (*N*=7).

**Table 3.** Fitness-genes predicted in *E. coli* UTI89 during growth in human urine

Locus_tag	Gene_name	Product	Main biological process (COG)	LogFC
UTI89_C1551	*yciM*	Lipopolysaccharide assembly protein B	LPS biosynthesis (G)	−6.95
UTI89_C2895	*rpoE*	RNA polymerase sigma E	Stress response/Transcription (K)	−6.77
UTI89_C0902	*cydD*	ATP-binding/permease protein CydD	Transmembrane transport/homeostasis (P)	−5.88
UTI89_C4289	*atpH*	Membrane-bound ATP synthase F1 sector delta-subunit	ATP synthesis (C)	−5.71
UTI89_C4171	*rfaJ* **^4^**	Lipopolysaccharide 1,2-glucosyltransferase	LPS biosynthesis (M)	−4.53
UTI89_C3837	*UTI89_C3837*	Hypothetical protein	(NI)	−4.43
UTI89_C3666	*degS* ^3^	Serine endoprotease DegS	Protease (O)	−4.36
UTI89_C3836	*rplA*	50S ribosomal protein L1	Ribosomal assembly/translation (J)	−4.36
UTI89_C4165	*rfaF* ^1^	ADP-heptose-LPS heptosyltransferase 2	LPS biosynthesis (M)	−4.32
UTI89_C3057	*csrA*	Carbon storage regulator	Regulation translation/transcription/ carbohydrate metabolism/biofilm (J)	−4.24
UTI89_C4292	*atpE*	Membrane-bound ATP synthase F0 sector subunit c	ATP synthesis (C)	−4.01
UTI89_C4290	*UTI89_C4290*	Hypothetical protein	(NI)	−3.94
UTI89_C3488	*hldE*	Bifunctional protein HldE	Carbohydrate metabolism (F)	−3.87
UTI89_C2309	*rfbC*	dTDP-4-dehydrorhamnose 3,5-epimerase	LPS biosynthesis (M)	−3.55
UTI89_C4291	*atpF*	ATP synthase subunit b	ATP synthesis (C)	−3.38
UTI89_C2825	*guaA*	GMP synthase (glutamine-hydrolyzing)	Purine metabolism (F)	−3.29
UTI89_C1262	*UTI89_C1262*	tRNA-specific 2-thiouridylase MnmA	tRNA modification (J)	−3.28
UTI89_C3660	*rpsI*	30S ribosomal protein S9	Translation (J)	−3.27
UTI89_C3492	*cca*	multifunctional CCA protein	RNA repair (J)	−3.19
UTI89_C4288	*UTI89_C4288*	Hypothetical protein	(NI)	−3.13
UTI89_C4287	*atpA*	ATP synthase subunit alpha	ATP synthesis (F)	−3.12
UTI89_C4379	*corA*	Magnesium transport protein CorA	Cobalt, magnesium and nickel transport (P)	−3.11
UTI89_C4286	*atpG*	ATP synthase gamma chain	ATP synthesis (C)	−3.04
UTI89_C4521	*UTI89_C4521*	Hypothetical protein	(NI)	−3.01
UTI89_C1260	*purB* ^1^	Adenylosuccinate lyase	Purine metabolism (F)	−2.87
UTI89_C4174	*rfaG* ^2^	Lipopolysaccharide core biosynthesis glucosyltransferase	LPS biosynthesis (M)	−2.84
UTI89_C4777	*purA* ^1^	Adenylosuccinate synthetase	Purine metabolism (F)	−2.79
UTI89_C4293	*atpB*	ATP synthase subunit a	ATP synthesis (C)	−2.78
UTI89_C4285	*atpD*	ATP synthase subunit beta	ATP synthesis (F)	−2.73
UTI89_C0657	*ybeY*	Endoribonuclease YbeY	rRNA processing/ribosome biogenesis (J)	−2.71
UTI89_C2852	*iscS*	Cysteine desulfurase IscS	Iron-sulphur cluster biosynthesis (E)	−2.65
UTI89_C3221	*recB*	RecBCD enzyme subunit RecB	DNA repair (L)	−2.62
UTI89_C0728	*UTI89_C0728*	Hypothetical protein	(NI)	−2.59
UTI89_C4166	*rfaC*	Lipopolysaccharide heptosyltransferase 1	LPS biosynthesis (M)	−2.58
UTI89_C4804	*UTI89_C4804*	HTH cro/C1-type domain-containing protein	DNA binding (K)	−2.57
UTI89_C4349	*wzxE* ^2^	Lipid III flippase	LPS biosynthesis (U)	−2.57
UTI89_C0022	*nhaA* ^2^	Na(+)/H(+) antiporter NhaA	Sodium transport and homeostasis/cation stress/regulation of intracellular pH (P)	−2.54
UTI89_C2031	*prc*	Tail-specific protease	Proteolysis/response to antibiotics (M)	−2.54
UTI89_C0729	*cydB*	Cytochrome bd-I ubiquinol oxidase subunit 2	Aerobic respiration/oxidative phosphorylation (C)	−2.53
UTI89_C0735	*tolA* ^2^	Membrane spanning protein TolA	Protein transport/cell division/membrane integrity (U)	−2.51
UTI89_C4945	*UTI89_C4945*	Hypothetical protein	(NI)	−2.43
UTI89_C3601	*yhbC*	Ribosome maturation factor RimP	Ribosomal biogenesis (J)	−2.42
UTI89_C3111	*rpoS*	Sigma factor RpoS	Stress response/transcription (K)	−2.41
UTI89_C5068	*dnaT*	Primosomal protein 1	DNA replication (J)	−2.41
UTI89_C3223	*recC* ^1^	RecBCD enzyme subunit RecC	DNA repair (L)	−2.39
UTI89_C1432	*galU* ^1,3^	UTP--glucose-1-phosphate uridylyltransferase	Galactose and UDP-glucose metabolism/ LPS biosynthesis (M)	−2.31
UTI89_C0734	*tolR*	Tol-Pal system protein TolR	Cell cycle/cell division (U)	−2.26
UTI89_C3154	*relA* ^1^	GTP pyrophosphokinase	Purine metabolism/response to starvation (F)	−2.25
UTI89_C4164	*rfaD*	ADP-l-glycero-d-manno-heptose-6-epimerase	Carbohydrate metabolism (G,M)	−2.24
UTI89_C4459	*glnA*	Glutamine synthetase	Nitrogen utilization/ammonia assimilation (F)	−2.24
UTI89_C0733	*tolQ*	Tol-Pal system protein TolQ	Cell cycle/cell division (U)	−2.24
UTI89_C2304	*wbdM*	Putative glycosyltransferase WbdM	LPS biosynthesis (M)	−2.23
UTI89_C1022	*ompA* ^3^	Outer membrane protein A	Ion transport/host-virus interaction/conjugation (M)	−2.20
UTI89_C3110	*UTI89_C3110*	Hypothetical protein	(NI)	−2.19
UTI89_C3986	*sira (tusA)*	Possible RNA-binding protein required for wild-type FtsZ ring formation on rich media	tRNA processing (J)	−2.18
UTI89_C2311	*rfbD*	dTDP-4-dehydrorhamnose reductase	LPS biosynthesis (F)	−2.12
UTI89_C2826	*guaB*	Inosine-5'-monophosphate dehydrogenase	Purine metabolism (F)	−2.11
UTI89_C1543	*topA*	DNA topoisomerase 1	DNA topological change (L)	−2.11
UTI89_C2026	*mgrB*	PhoP/PhoQ regulator MgrB	Transcription regulation/response to Mg2 +ion (S)	−2.05
UTI89_C0736	*tolB*	Tol-Pal system protein TolB	Protein transport/cell cycle, cell division (U)	−2.03
UTI89_C2053	*eda*	KHG/KDPG aldolase	Carbohydrate metabolism (G)	−2.01
UTI89_C1266	*icdA*	Isocitrate dehydrogenase	Tricarboxylic acid cycle (C)	−2.01
UTI89_C1265	*UTI89_C1265*	Hypothetical protein	(NI)	−2.01
UTI89_C0016	*dnaK*	Chaperone protein DnaK	DNA replication/stress response (O)	−2.00

Genes found as fitness-genes during infection of the mouse bladder (list corresponding to composite sample UTI89_Z) are underlined (LogFC < −2). NI: not identified. Ambiguous- and not fitness-genes are depicted in Table S5.

Gene products were re-annotated using the UniProt database.

Cut-off for selection of fitness-genes: LogFC < −2, *Q*<0.001

^1^Genes previously also identified as important for growth in human urine in UPEC CFT073 using a Tn-ordered library [38].

^2^Genes previously also identified as required for serum resistance of UPEC EC958 using TraDIS [21]

^3^Genes relevant for UTI and/or virulence of UPEC reported by other studies [43, 65, 72]

^4^Gene relevant for growth in urine after one passage.

A previous study using an ordered Tn-library in UPEC CFT073 detected 87 fitness-genes during growth in human urine [38]. Of them, one gene (c2450, encoding a hypothetical protein) was absent from the UTI89 genome, and four genes were found in the list of urine fitness-genes for UTI89 in the current study (Table 3). The gene *yciM*, encoding a tetratricopeptide repeat protein required for regulation of LPS biosynthesis [39] showed the greatest difference between the output and input libraries (logFC of −6.9) (Table 3).

Urine fitness-genes were categorized according to function. The most commonly identified functions in UTI89 were genes encoding hypothetical proteins (no COG assigned) (12.5%), associated with LPS biosynthesis (15.6%), involved in ATP synthesis (10.9 %), involved in purine biosynthesis (6.25%), encoding factors of the Tol-Pal system (6.25%) and encoding ribosomal proteins (6.25%) (Table 3). The remaining genes (41.25%) were classified as virulence genes, membrane proteins, transcription and translation regulators, metabolic functions (for example, carbohidrate metabolism), cell cycle and division, replication, ion transport, DNA and RNA repair, etc. The genes *purA, purB* and *guaA,* involved in purine biosynthesis [40] were detected as fitness-genes UTI89 (Tables 3 and S5). These genes have previously been revealed as important for growth of UPEC in human urine [38, 41]. Interestingly, two genes, *rfaG,* LPS biosynthesis related, and *tolA,* belonging to the Tol-Pal system, have previously been confirmed as required for serum resistance in UPEC EC958 [21]. LPS biosynthesis was previously suggested to be relevant for UPEC growth and survival in urine from women with UTI, with the gene *rfaG,* among others*,* found to be highly expressed in UPEC during growth in this medium [7]. In a recent work, the Tol-Pal system was found to be required for optimal internalization, aggregation followed by IBC formation within urinary tract cells, as well as for bacterial motility in CFT073 [42]. The *ompA* gene, encoding an outer membrane porin, detected as fitness-gene in UTI89, was previously identified as a putative UPEC fitness-gene in human urine [43]. No fimbria or iron-related genes were identified as fitness-genes during growth in human urine in the current study.

### *E. coli* UTI89 fitness-genes during mouse cystitis

Mice produce highly concentrated urine, and high urine concentrations have been shown to affect growth and induce stress responses such as filamentation in UPEC [25, 44]. This could also influence the bottleneck effect, which has previously been described as a potential problem when assaying high-complex Tn-libraries in animal infection experiments [45]. Therefore, in the current study mice were fed with 5 % glucose in water for 16 h prior to infection as reported [18, 19]. This promotes UTI, causes mild diuretic effects and lowers the urine concentration to reach the concentration normally found in humans; a significant reduction (*P*=0.04) in USG from 1.06±0.002 (pre-treatment with glucose) to 1.05±0.009 (after 16 h treatment with glucose) was observed. These mice were inoculated with approximately 4.5×10^7^ c.f.u. and successful colonization of murine bladders was observed (Table S1). Thus, with this approach, approximately 10^5^–10^6^ c.f.u. ml^−1^, corresponding to 1–10 times the size of the UTI89 input library, were obtained from two individual bladder samples, as well as from a composite sample (Table S1). Output libraries from the two single mice (UTI89_V and UTI89_W) as well as an output library obtained by mixing libraries corresponding to 11 mice (UTI89_Z) were processed for further TraDIS analysis. Tn-insertions were detected every 54.2–130 bp in these samples (Table 2) indicating a lower saturation than in the input library. A total of 579 genes were identified as fitness-genes for bladder colonization based on the composite library (UTI89_ Z) (Tables 4 and S6). At the single mouse level, 607 (UTI89_V) and 1244 (UTI89_ W) (Table S6) fitness-genes were identified, suggesting large mouse to mouse variation, and stressing the need to perform the analysis based on composite samples. Of the fitness-genes identified, 50 were included in the list of genes required for bacteriuria (Fig. 4c). A total of 367 fitness-genes were common to all the output libraries (UTI89_V, UTI89_W and UTI89_Z) (Fig. 4b, Table S6). As shown for urine, genes involved in LPS biosynthesis, ATP synthesis and members of the Tol-Pal system were found to be fitness-genes for bladder infection (Tables 4 and S6). Contrary to what was observed during growth in urine, fimbria- (*auf*, *yeh* and *fim*), iron (*fur*) and amino acid-related genes (*artJ, metQ*) were identified as fitness-genes during bladder infection (Table S6). In a previous study based on and ordered Tn-mutagenesis in UPEC CFT073 [38], 36 genes were found as potentially relevant for colonizing the bladder at 16 h p.i. One of the fitness-genes identified in CFT073, *c2497,* was not present in the genome of UTI89. Seven of the genes, *efp*, *rfaG*, *ftsJ*, *purB, fimG*, *fimH* and *ldcA* were also detected in the current study (Tables 4 and S6). A total of 31 genes were shared by the five UTI89 output libraries analysed in this study: mice (UTI89_Z), urine (20 and 40 h), EZ-MOPS and LB (Fig. 4d, Table S6).

**Table 4.** Top 50 fitness-genes predicted in *E. coli* UTI89 during mouse bladder colonization showing the greatest LogFC compared to the input library

Locus_tag	Gene_name	Product	Main biological process (COG)	LogFC*
UTI89_C4380	*UTI89_C4380*	Conserved hypothetical protein	(S)	−12.53
UTI89_C4865	*holC*	DNA polymerase III, chi subunit	DNA replication (L)	−12.33
UTI89_C0735	***tolA***	Membrane spanning protein TolA	Protein transport/cell division/membrane integrity (U)	−12.27
UTI89_C0060	*surA*	Chaperone SurA	Protein folding/stabilization (O)	−12.26
UTI89_C4165	***rfaF***	ADP-heptose-LPS heptosyltransferase 2	LPS biosynthesis (M)	−12.26
UTI89_C3666	***degS***	Serine endoprotease DegS	Protease (O)	−12.04
UTI89_C3223	***recC***	RecBCD enzyme subunit RecC	DNA repair (L)	−11.94
UTI89_C3978	*ftsE*	Cell division ATP-binding protein FtsE	Cell division/cell cycle (D)	−11.46
UTI89_C4166	***rfaC***	Lipopolysaccharide heptosyltransferase 1	LPS biosynthesis (M)	−11.40
UTI89_C1134	*UTI89_C1134*	Hypothetical protein	(NI)	−11.20
UTI89_C4332	*rep*	ATP-dependent DNA helicase Rep	DNA replication/DNA repair (L)	−11.15
UTI89_C2716	*ypdE*	Aminopeptidase YpdE	Protease (G)	−10.98
UTI89_C1905	*himA*	Integration host factor subunit alpha	Conjugation/DNA recombination (K)	−10.98
UTI89_C2382	*yehA*	Putative Yeh fimbiral adhesin YehA	Cell adhesion (N,U)	−10.89
UTI89_C2825	***guaA***	GMP synthase (glutamine-hydrolyzing)	Purine metabolism (F)	−10.88
UTI89_C1527	*yciF*	Putative structural protein	DNA damage (S)	−10.74
UTI89_C2232	*UTI89_C2232*	Hypothetical protein	(NI)	−10.73
UTI89_C2309	***rfbC***	dTDP-4-dehydrorhamnose 3,5-epimerase	LPS biosynthesis (M)	−10.65
UTI89_C3163	*syd*	Protein Syd	Regulation protein assembly (S)	−10.63
UTI89_C1535	*yciO*	YrdC-like domain-containing protein	RNA binding (J)	−10.52
UTI89_C4945	***UTI89_C4945***	Hypothetical protein	(NI)	−10.40
UTI89_C1711	*ydeM*	Anaerobic sulfatase maturase	Metal binding (C)	−10.40
UTI89_C2304	***wbdM***	Putative glycosyltransferase WbdM	LPS biosynthesis (M)	−10.31
UTI89_C0983	*himD*	Integration host factor subunit beta	Conjugation/DNA recombination (K)	−10.31
UTI89_C0170	*hemL*	Glutamate-1-semialdehyde 2,1-aminomutase	Protoporphyrin-IX biosynthesis (H)	−10.28
UTI89_C0739	*ybgF*	Cell division coordinator CpoB	Cell division (D)	−10.21
UTI89_C1213	*yceD*	23S rRNA accumulation protein YceD	Synthesis, processing and/or stability of 23S rRNA (S)	−10.16
UTI89_C3977	*ftsX*	Cell division protein FtsX	Cell division/cell cycle (D)	−10.11
UTI89_C2256	*UTI89_C2256*	Hypothetical protein	(NI)	−10.07
UTI89_C3847	*yheN*	Sulfurtransferase TusD	tRNA-processing (J)	−10.05
UTI89_C4164	***rfaD***	ADP-l-glycero-d-manno-heptose-6-epimerase	Carbohydrate metabolism (G,M)	−10.05
UTI89_C4901	*UTI89_C4901*	Hypothetical protein	(NI)	−10.04
UTI89_C4734	*cutA*	Divalent-cation tolerance protein CutA	Response to copper (P)	−10.04
UTI89_C0145	*UTI89_C0145*	Hypothetical protein	(NI)	−9.93
UTI89_C4688	*phnO*	Putative regulator, phn operon	Aminophosphonate metabolic process (K)	−9.93
UTI89_C3228	*UTI89_C3228*	Hypothetical protein	(NI)	−9.91
UTI89_C2384	*yehC*	Putative periplasmic chaperone YehC	Cell wall organization/protein folding (N,U)	−9.90
UTI89_C5118	*UTI89_C5118*	Hypothetical protein	(NI)	−9.88
UTI89_C1526	*yciE*	DUF892 domain-containing protein	(S)	−9.88
UTI89_C3488	***hldE***	Bifunctional protein HldE	Carbohydrate metabolism (F)	−9.82
UTI89_C4745	*efp* ^†^	Elongation factor P	Translational elongation (J)	−9.78
UTI89_C4031	*UTI89_C4031*	Hypothetical protein	(NI)	−9.76
UTI89_C2892	*rseC*	Sigma-E factor regulatory protein RseC	Oxidative stress (T)	−9.67
UTI89_C1550	*yciS*	Lipopolysaccharide assembly protein A	LPS biosynthesis (S)	−9.62
UTI89_C0088	*UTI89_C0088*	Hypothetical protein	(NI)	−9.62
UTI89_C0750	*UTI89_C0750*	Putative homeobox protein	DNA binding (S)	−9.57
UTI89_C2839	*UTI89_C2839*	Hypothetical protein	(NI)	−9.55
UTI89_C2840	*ndk*	Nucleoside diphosphate kinase	Purine and pyrimidine metabolism (F)	−9.55
UTI89_C4612	*dgkA*	Diacylglycerol kinase	Phosphatidic acid biosynthetic process (M)	−9.53
UTI89_C4083	*UTI89_C4083*	Hypothetical protein	(NI)	−9.50

*LogFC values shown are those detected for the genes in output library Z (representing all 11 mice). The remaining significantly affected genes together with ambiguous- and not fitness-genes are depicted in Table S6.

†Gene previously found as the potential fitness-gene for bladder colonization in UPEC CFT073 using an ordered Tn-library [38].

Genes also found to be relevant for growth in human urine in the current study are highlighted in bold.

Gene products were re-annotated using the UniProt database

Cut-off for selected fitness-genes in mice: LogFC < −2 and *Q*<0.001.

Contrary to growth in human urine where we did not find any anti-fitness-gene, 147 potential anti-fitness-genes were detected during mouse bladder colonization (Table S6).

### Validation of fitness-genes for growth in human urine

Genes that were identified as fitness-genes during growth in urine, and which were not previously confirmed as relevant for growth in this niche in UPEC were selected for further validation using deletion-mutants. A total of 19 mutants were constructed in the UTI89 strain including 17 single mutants, a double mutant for the genes *rfaD* and *rfaC* and a mutant lacking the *tol* operon. Mutant strains were tested for the ability to grow in human urine in single or competition assays, and for comparison in EZ-MOPS, and LB (Table 1). Whole-genome sequencing and bioinformatic analysis confirmed that all the mutants lacked the expected specific genes, which were replaced with a selectable antibiotic resistance marker, and that no additional mutations were present in the strains compared to the WT UTI89. Therefore, every phenotype observed for the mutant could unambiguously be related to the site-specific mutations. In addition, results from competition experiments in LB, EZ-MOPS and human urine confirmed that neither the rifampicin nor the chloramphenicol resistance did imply any additional cost for the bacterium since no significant differences in growth were observed between the strains tested (not shown).

**Fig. 5.**
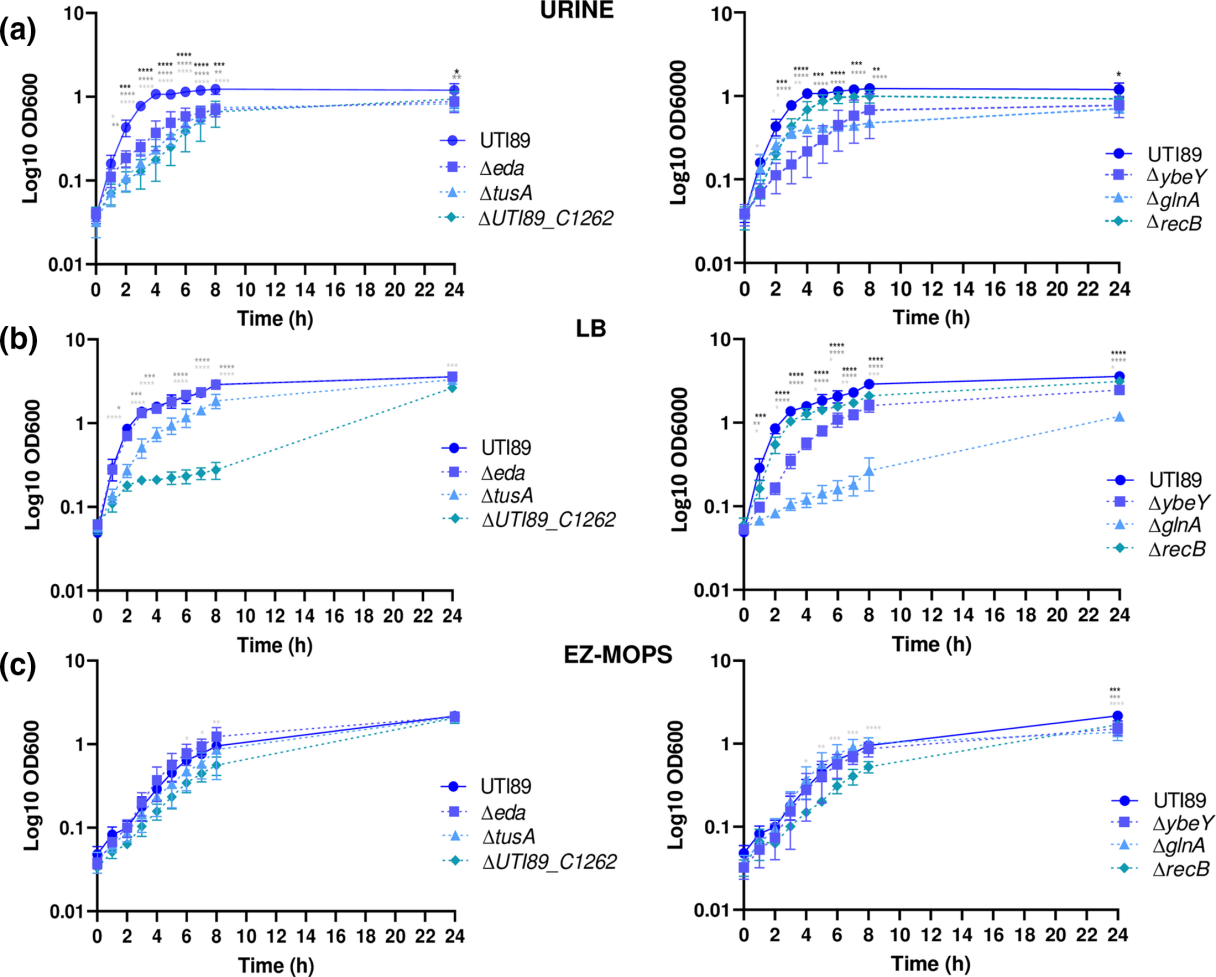
Growth curves obtained for *E. coli* UTI89 and its mutant derivatives in urine (a), LB (b) and EZ-MOPS (c). The data shown are means±standard deviations of three biological replicates. Statistical significance (****P < 0.0001; ***P < 0.001; **P < 0.01; *P < 0.05) was determined by one-sample t-test or ANOVA and Sidak’s post-test. Black, dark and light grey asterisks show significant differences between *E. coli* UTI89 and the mutants (∆*eda* or ∆*ybeY*, ∆*tusA* or ∆*glnA* and ∆*UTI89_C1262* or ∆*recB*), respectively. Only results for validated genes in urine are shown.

**Fig. 6.**
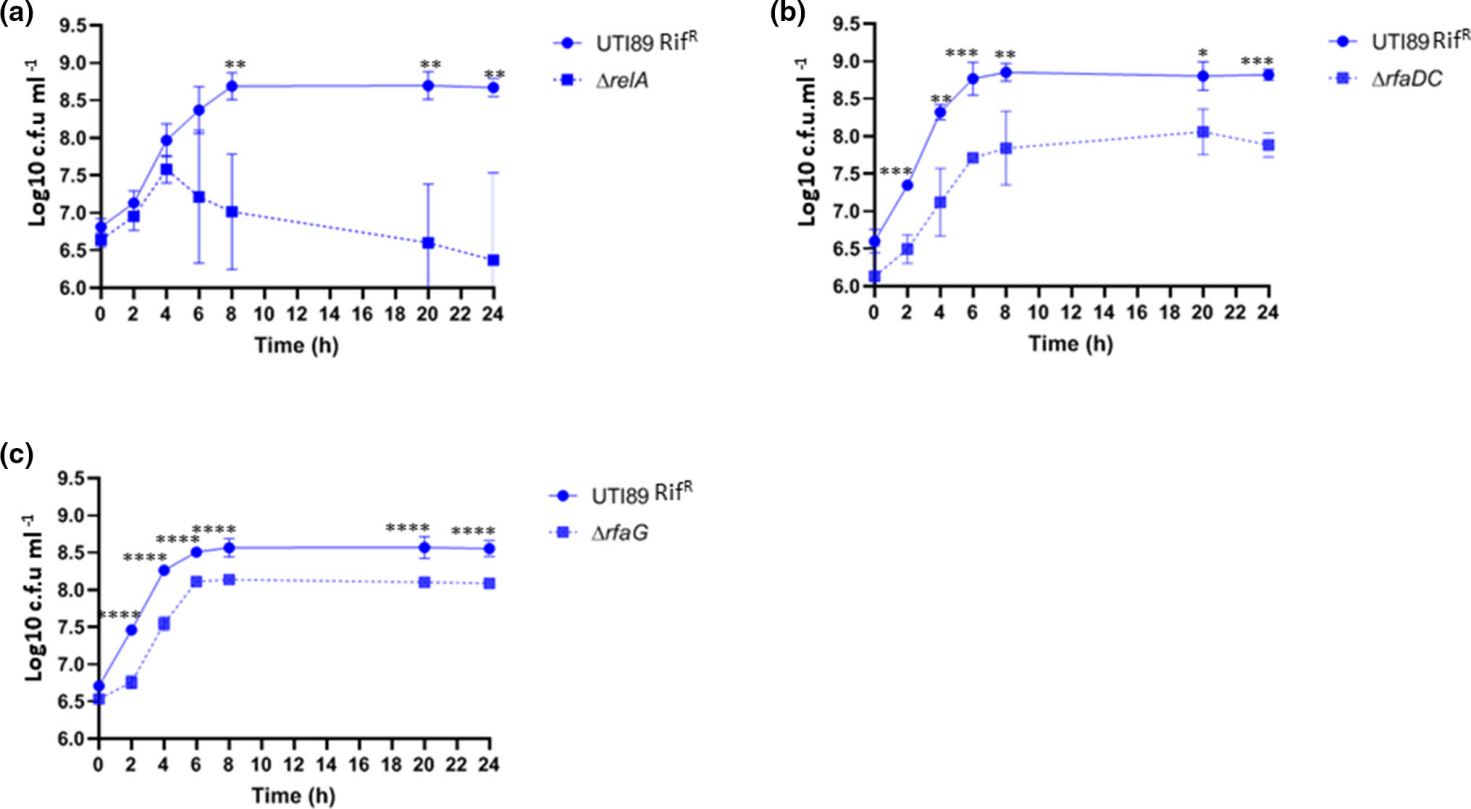
Competition assays of the *E. coli* UTI89 rifampicin resistant strain (UTI89 Rif^R^) and the mutants Δ*relA* (a), Δ*rfaDC* (b) and Δ*rfaG* (c) in human urine. The data shown are means±standard deviations of at least three biological replicates. Statistical significance (****P< 0.0001; ***P< 0.001; **P< 0.01; *P < 0.05) was determined by one-sample *t*-test at each time point tested. Only results for validated genes are shown.

Nine mutants, namely, *∆eda*, *∆tusA, ∆UTI89_C1262, ∆ybeY, ∆glnA, ∆recB, ∆relA, ∆rfaDC* and *∆rfaG* showed significant growth attenuation compared to the WT UTI89 during growth in urine (Figs 5 and 6). For *∆relA*, *∆rfaDC* and *∆rfaG*, the difference was only significant when competition experiments were performed (Fig. 6). The fact that these genes showed significantly reduced score (LogFC < −2) in the output library in urine compared to the input library as revealed by TraDIS combined with a significant growth defect of site-specific mutants in the medium qualify them as validated urine fitness-factors. Five of them, *UTI89_C1262, ybeY, glnA, recB* and *rfaDC* were also attenuated during growth in EZ-MOPS and LB (Fig. 5), while *tusA* was only found to affect growth in urine and LB (Fig. 5). Interestingly, none of the six genes detected by TraDIS and verified as important for growth in LB were identified as part of the list of fitness-genes in *E. coli* K-12 BW25113 during growth in this medium [35]. The remaining mutants (*n*=10) showed similar growth patterns as UTI89 in urine, EZ-MOPS and LB (Figs S1 and S2), except the mutant Δ*prc,* which was significantly attenuated for growth in LB and EZ-MOPS and the mutant Δ*tol,* which showed a significant growth defect in EZ-MOPS (Fig. S1). All genes verified to affect fitness of UTI89 in EZ-MOPS and LB had initially been classified as fitness-genes for the corresponding growth condition, except *UTI89_C1262* in EZ-MOPS (Table S5). Some of the genes, which were not validated to cause growth attenuation in urine, i.e. the tol-Pal system and *ompA* (Figs S1 and S2), were previously demonstrated to be involved in other stages of UTI [42, 46]. In a previous Tn-mutagenesis-based study using the strain CFT073 [38], mutants for eight genes; *yigP, ubiG*, *ftsJ, gidA, thdF*, *ldcA, purB* and *carB*, showed a visible fitness defect during growth in human urine. Among these, only *purB* was identified as a fitness-gene for growth in urine using our TraDIS approach (Table 3), but since it was confirmed as important for growth in human urine in CFT073 [38], the gene was discarded for further validation in our study. The remaining genes previously identified in CFT073 showed the following LogFC; *yigP*: 0.26, *ubiG*: −0.78, *ftsJ*: −1.89, *gidA*: −1.52, *thdF (trmE* in UTI89)*:* −1.3*, ldcA*: −0.29, and *carB*: −1.47 and *Q*<0.01 for all of them.

### Validation of fitness-genes during mouse UTI

Eleven out of the 579 fitness-genes identified from the composite library (UTI89_Z) (Table 4) were selected for site-specific mutagenesis and further validation in the mouse model of UTI using a co-challenge approach (Table 5). Criteria for gene selection included high LogFC values (< −7) and no previous confirmation of a role during infection of the murine UTI model. Growth rates of the mutants were shown not to differ significantly from the growth rate of the WT *in vitro* in LB (data not shown). Also here, sequencing and further analysis of the mutants confirmed specific deletion of genes without additional mutations in the strains. Results showed that 10 out of 11 mutants were outcompeted by the WT with competitive indexes ranging between 0.003 and 0.500 (Table 5). The confirmed fitness-genes encoded functions related to LPS biosynthesis (*rfaG*), ATP synthesis (*atpF*), copper tolerance (*cutA*), magnesium uptake (*corA*), DNA recombination (*himD*), polysaccharide transport (*wzxE*), cell division (*ftsE*) and several metabolic pathways (*phnO, ypdE* and *tam*) (Table 4). The *sufA* mutant did not show significant reduction in infection potential (CI of 1.493) (Table 5). In the previous study using CFT073, seven Tn-mutants in the genes *yihE*, *purB, carB*, *yfgM*, *rfe*, *efp* and *flhB* showed a fitness defect compared to the WT during colonization of the mouse bladder, using a 16 h co-challenge assay [38]. Of them, *efp* and *purB* were predicted as important for bladder colonization in the current study (Table S6) while the remaining genes were not included in our lists of fitness-genes. This encompassed *yhiE* with a LogFC of −0.18, *carB* (−0.65), *ygfM* (−0.66) and *flhB* (−0.40), however, all with a *Q* value<0.01.

**Table 5.** Competitive indexes for *E. coli* UTI89 mutants in mice

* E. coli* strains (no. of mice)	Competitive index (CI)
	Bladder
UTI89 Rif^Ra^	
Versus	
*ΔcorA* ^b^ (six mice)	0.268 ± 0.135^c^
*ΔwzxE* ^b^ (six mice)	0.377 ± 0.210^c^
*ΔftsE* (six mice)	0.027 ± 0.034^c^
*ΔypdE* (six mice)	0.297 ± 0.236^c^
*ΔcutA* (six mice)	0.332 ± 0.134^c^
*ΔatpF* ^b^ (six mice)	0.402 ± 0.298^c^
*ΔrfaG* ^b^ (five mice)	0.500 ± 0.252^c^
*ΔhimD* (five mice)	0.003 ± 0.002^c^
*Δtam* (six mice)	0.325 ± 0.194^c^
*ΔsufA* (six mice)	1.493±0.759
*ΔphnO* (five mice)	0.476 ± 0.401^c^

^a^A rifampicin-resistant derivative mutant of WT UTI89 was used in the assays.

^b^Genes also predicted as UTI89 fitness-genes for growth in human urine although only *rfaG* was confirmed to be important during growth in that medium. All mice in each group survived to the end of the experiment. CIs were estimated on the basis of the c.f.u. ml^-1^ of inoculum and c.f.u. ml^-1^ detected for each suspension of bladders of the mutants compared to the same values for WT as previously described [34]. The results are shown as mean values±standard deviations for the total number of mice tested (indicated in parentheses in the first column). Statistical significance was determined by one-way ANOVA with Dunnett´s multiple comparison test.

^c^CI was significantly different from 1, *P*<0.05.

## Discussion

The success of a bacterial pathogen entails a balance between fitness and the need to express highly energy-demanding virulence factors that contribute to growth and survival in the host [47]. Moreover, the required set of fitness and virulence genes might be strain- and/or host-dependent [7–11]. In this study, we used TraDIS, a technique combining Tn-mutagenesis and sequencing to characterize relevant growth factors in the commonly used UPEC strain UTI89; this included identifying essential-genes for *in vitro* growth on LB agar plates as well as genes contributing to fitness during growth in frequently used lab media, human bacteriuria and mouse cystitis. Thus, the study provides basic knowledge on the fitness landscape of this well-known UPEC strain in the laboratory as well as during two relevant stages of UTI.

The gene essentiality analysis revealed differences in the essential-genes detected in UPEC UTI89 when compared to *E. coli* K-12 BW25113 and UPEC EC958 [21, 35], where TraDIS was applied under similar conditions. However, in spite of the differences observed related to single genes, the COG analysis showed that similar functions were essential for growth in the two UPEC strains. These results indicate that a specific *E. coli* strain displays a particular set of essential-genes for growth, but at the functional level they might belong to the same categories in different isolates. The comparative results should be analysed in more detail, and further validation studies might be performed to confirm gene dispensability for growth in the different strains. It should be noted that TraDIS may overlook essential-genes if only one domain in the encoded protein is essential, and the encoding part is not hit by the transposon. In the current study, insertions were identified every approximately 20 bp, making this unlikely. Also, the genetic discrepancies found compared to previous studies may be due to differences in library sizes; those constructed in K-12 BW25113 and EC958 were larger (approximately 3.7 and 1 million mutants, respectively) [21, 35], and thus more saturated than the one generated here, which may lead to differences when genes are statistically scored as essential. In the mentioned studies, genes were considered essential at LLR <−3.6 [21, 35], while here a gene was categorized as essential if showing a LLR < −2. Finally, differences in the genome content; i.e. some genes present in UTI89 but absent in other *E. coli* strains, and vice versa, might explain the diverse repertoires of essential-genes obtained.

LB is widely used as a growth medium rich in nutrients because of its convenience and high bacterial growth yield [36]. However, variability is a recognized problem due to the ill-defined nature of the major components [48, 49]. To be able to define *in vitro* fitness-genes in this media as well as under more precise growth conditions (i.e. available nutrients are known), we tested the input library during growth in both LB and EZ-MOPS, which is a rich well-defined medium, that also allows great bacterial yield, and is suitable for studies where it is relevant to acknowledge the components of the medium [48]. Results showed that the *E. coli* UTI89 fitness-gene-repertoire differed slightly between growth in LB and growth in EZ-MOPS with a higher number of fitness-genes identified during growth in EZ-MOPS (*N*=126 versus *N*=73), however, the pool in EZ-MOPS may contain essential-genes for growth in this medium as well. Overall, this result may indicate fewer nutritional requirements for *E. coli* during growth in LB compared to EZ-MOPS. Our results point to the importance of carefully selecting the appropriate medium depending on the microbiological study performed. Interestingly, our list of UTI89 fitness-genes in LB differed from the equivalent one obtained in *E. coli* K-12 using TraDIS (73 versus 356 genes identified) suggesting that differences in genetic requirements for growth in this medium are strain-dependent. However, the statistical approach used for detection of fitness-genes in K12 [35] differed from the one applied in our study, which may explain some differences observed.

Recently, gene essentiality in 18 *E. coli* strains, including UTI89, was assessed and compared during growth in LB, M9-glucose and gut microbiota media using a novel CRISPR interference screening platform [50]. A total of 340 essential genes for growth in LB in UTI89 were identified, which disagrees with the current study (73 genes detected). Notably, discrepancies might be explained by the different approach used and the screening conditions performed.

Growth of UPEC in human urine has frequently been used as a useful approach to detect bacterial genes and proteins of relevance for UTI [41, 51–54]. Urine has been described as a medium where certain nutrients such as nitrogen and iron are scarce, and bacteria have to adapt their metabolism in order to overcome the limitations and survive [52]. In general, the urinary tract is characterized by being a high-osmolarity, moderately oxygenated, iron-restricted environment containing mostly amino acids and small peptides [55]. Interestingly, our results revealed a set of fitness-genes during growth in human urine for UTI89, which differed from this obtained for other UPEC strains tested in previous studies [38, 43, 54, 56], suggesting that genetic requirements during growth in the medium are strain-dependent and/or vary depending on the experimental set up and method used to score fitness. Thus, neither peptide transporters- nor amino acid biosynthesis-related genes were fitness-genes for UPEC UTI89 during growth in urine. This contradicts previous work [38, 43, 54, 56], although, others have shown that mutants, which are auxotrophic for arginine and serine biosynthesis did not display growth defects during UTI [43]. Notably, the gene *relA*, associated with a stringent response induced, for example, by amino acid starvation, was proved to be a fitness-factor in UTI89 during growth in human urine, and several genes related to amino acid metabolism and peptide transport were classified as ambiguous in our work. Likewise, we did not detect genes involved in the transport and catabolism of sialic acid, gluconate, xylose and arabinose in the pool of fitness-genes in urine, despite their reported high expression in UPEC cultured in human urine [43, 57]. Interestingly, sulphur-acquisition might be of relevance during growth in human urine since *tusA* encoding a sulphur carrier protein, which provides sulphur for different metabolic pathways [58] was confirmed to be important for UTI89 growth in this niche. No genes related to iron metabolism (uptake or transport) were found as fitness-genes during growth in urine, which seems to contradict previous studies suggesting that UPEC struggles to obtain iron during growth in this environment [43, 52, 53]. However, several genes involved in iron uptake and storage, such as *iroN* or *ftnA,* were classified as ambiguous by our TraDIS approach in urine, and further, UPEC has several overlapping systems to obtain iron [3], making the individual genes dispensable. Also, the extent of iron limitation may differ among donors as suggested [7]. Cell adhesion factors were not identified during growth in human urine either, in disagreement with previous studies [10]. The glycolitic pathway, Entner–Doudoroff (ED), was also confirmed as important during growth in human urine, since the mutant lacking the *eda* gene, involved in the degradation of glucose via the ED pathway showed a fitness defect compared to the WT during growth in this medium.

The UTI89 fitness-gene-set during mouse colonization of the bladder was likewise analysed by TraDIS. In our model, infection time was 6 h, mainly leading to the identification of factors involved in early colonization of the bladder. In order to overcome, at least partially, the limitations of the bottleneck effect associated to the traditional model of UTI when performing Tn-insertion sequencing studies [45], we used a well-established mouse model of ascending UTI with some modifications [18, 19, 24]. In the model applied here, mice were administered glucose in drinking water for 16 h prior to infection to cause mild diuresis and consequently lower the urine concentration. To note, we carried out preliminary similar experiments where the infection period was of 48 h and 6 h without glucose administration (unpublished). However, for the 48 h experiment, as revealed by the TraDIS analysis, we did not manage to cover our library size; some mutants appeared to be over-represented and others were probably arbitrary lost before they could colonize the bladder due to urination. Reducing the infection time to 6 h and maintaining the dose led to insufficient colonization of the bladder. Thus, glucose treatment was done, on the one hand to overcome the bottleneck limitation, and on the other hand to create a urine environment close to this observed in humans with a similar urine concentration [18, 19]. However, the fitness-factors identified based on analysis of single mouse bladders showed large variation between animals, with remarkable differences in UIS observed among them, suggesting that the bottleneck effect might still occur in single animals. Consequently, we chose to perform our output analysis based on a pooled sample representing the 11 mice. This may lower the risk that a gene is lost by chance, since this has to happen simultaneously in several mice before it affects the scoring significantly. A similar observation to the mouse-to-mouse variation here has been made in transcriptomic-based studies using human urine from women suffering from UTI, where different genes were up-regulated depending on the urine donor [7]. Importantly, when pooling bladder from mice, approximately 80 % of the fitness-genes identified in UPEC UTI89 for growth in human urine were also detected as putatively relevant for infection of the mouse bladder. This indicates that the fitness-genes observed in the current study represent indeed good candidates for fitness-genes during human UTI. A previous study [38] addressed the bottleneck effect by using an ordered Tn-mutant library, created in UPEC CFT073, reducing the number of mutants down to 9216 mutants, which was then tested in the standard mouse model of UTI [59].

Contrary to results in human urine, *fur*, involved in iron uptake and several fimbrial-related genes were found as relevant during mice bladder colonization. Interestingly, *corA* and *cutA* genes, involved in transport of magnesium and related to copper tolerance, respectively, were important for mouse bladder colonization, which suggests that magnesium is scarce while copper is highly abundant in the bladder environment. Contrary to previous studies, genes involved in the import of petides (*dppA* and *oppA*), gluconeogenesis (*pckA),* genes involved in the uptake of nickel (*nik* genes) or potassium (*kdp*), osmotic stress, putrescine biosynthesis (*speB*), copper tolerance (*cus*)*,* a gene encoding a formate dehydrogenase (*fdhF*), and different virulence genes (*cjrABC-senB* pUTI89-plasmid located-, and *fbp* and some genes belonging to UPEC genomic islands) were not scored as fitness-genes in the current study, despite the corresponding mutations being reported to lead to a fitness defect *in vivo* [9, 43, 57, 60, 61]. Notably, except for the genes pUTI89-associated, these genes were confirmed as relevant for bladder colonization in CFT073 and most of them were classified as ambiguous in this study. Capsule biosynthesis (*kps*) genes were predicted as relevant for bladder infection as previously shown for UPEC CFT073 [9]. Biofilm formation has been demonstrated to be relevant for mouse and pig bladder colonization [62, 63]. In accordance with this, the curli-related genes (*csr),* (also involved in gluconeogenesis and glycolysis), and *csgF* playing a role in motility and biofilm formation [62, 64], were identified as fitness-factors during colonization of the murine bladder. Other genes found as potentially relevant *in vivo* for the outcome of UTI in our study, such as *degS*, *surA* and *marA* encoding a serine protease, an isomerase and a multiple antibiotic resistance protein, respectively, have all been demonstrated to play a role during infection of mice [65–67]. As previously shown [9, 55], ethanolamine metabolism, particularly the gene *eutK,* was also predicted to be relevant for host colonization during UTI.

Genes associated to purine metabolism were revealed as fitness-determinants in both niches, which confirms previous observations [43, 68]. Overall, our TraDIS results combined with the outcome from infection with site-specific mutants showed the importance of glycolysis, ATP synthase, ribosomal proteins, oxidative phosphorylation and as previously confirmed the TCA [43] with the gene *icdA*, detected as part of the UTI89 fitness-gene-set in human urine and mice, during bacteriuria and mouse cystitis. Our results also support that the urinary tract is nitrogen-limited [7] with the gene *glnA* confirmed as a fitness-factor during growth in human urine and included in the lists of fitness-genes during mouse cystitis. In agreement with previous studies where the importance of motility/flagella during UTIs was discarded [51], flagella-related genes were not identified as contributing to fitness of UTI89. As reported, genes involved in LPS biosynthesis are important for UPEC to grow in the urinary tract [7, 21]. Thus, and of interest regarding selection of future targets for infection control, the gene *rfaG* was confirmed to be relevant for growth in human urine and for infection of the mouse bladder. This gene has previously been identified as a fitness-gene for UTI using different –omics approaches and in different UPEC strains [7, 21, 38, 69]. The Tol-Pal system that was previously proved to be relevant at different stages of the UTI [21] was also found to be important under both test conditions in the current study, according to TraDIS results, however, neither a mutant lacking the *tol* operon nor the *tolQ* gene showed attenuated growth in urine compared to WT. It should be noted that despite several attempts, the gene *tolA*, could not be deleted in the WT UTI89 strain, and thus the importance of this gene could not be investigated *in vivo*. Nevertheless, the gene has previously been mutated in other UPEC strains [21, 42], indicating a strain-specific character.

Other potential UTI89 fitness-genes during UTI included regulators, genes involved in DNA damage and repair and hypothetical proteins. Moreover, 8 and 187 genes encoding hypothetical proteins were identified as candidate fitness-genes of UTI89 in bacteriuria and mouse cystitis, respectively, and seven of them were found in both niches. It would be of interest to further investigate the role of such factors during UTI. Importantly, some of them might be membrane-located and may represent targets for new therapeutics and prophylactics. As previously found [38], we also observed potential redundancy of multiple fitness-genes (for example, ATP synthesis, LPS, cell division).

The relatively low confirmation rate of validated genes in urine (only approximately 50 % of the selected genes were confirmed to be relevant for growth in urine) might be explained by the fact that urine is a complex medium whose composition is unstable. Despite using urine from the same donors, urine nutrients might differ depending on the time point where urine was collected with factors such as changes in diet and fluid intake having an influence on it. Also, more donors should be tested to assess the variation related to host.

A limitation of using large pools of Tn-mutants to identify fitness-genes is that trans-complementation, where genetic defects of one mutant are compensated by the presence of the functional genes in other mutants, as previously discussed [47] may occur. Also, the Tn*5* transposon carries an outward facing promoter that drives expression of the Kn resistance gene and, as a result, individual Tn-insertions can cause polar effects due to the increased transcription of downstream genes [70, 71].

In conclusion, this work has identified and validated a number of novel fitness-genes in UPEC for growth in laboratory media and for causing UTI, all valuable information enabling us to understand the biology of UPEC during infection and how this differs from life in the laboratory media. The set of fitness-genes was further revealed to differ depending on the growth media. As part of the study, a modified mouse model of UTI was implemented, which allows for identification of fitness-genes when using complex Tn-libraries.

## Supplementary Data

Supplementary material 1

Supplementary material 2

Supplementary material 3

Supplementary material 4
