# Supplementary material for: Genome-wide analysis of fitness-factors in uropathogenic Escherichia coli during growth in laboratory media and during urinary tract infections
Source: Microb Genom. 2021 Dec 20;7(12):000719. doi: 10.1099/mgen.0.000719 (PMC8767336; doi:10.1099/mgen.0.000719)
Supplement: Supplementary material 1 [file mgen-7-0719-s001.pdf]

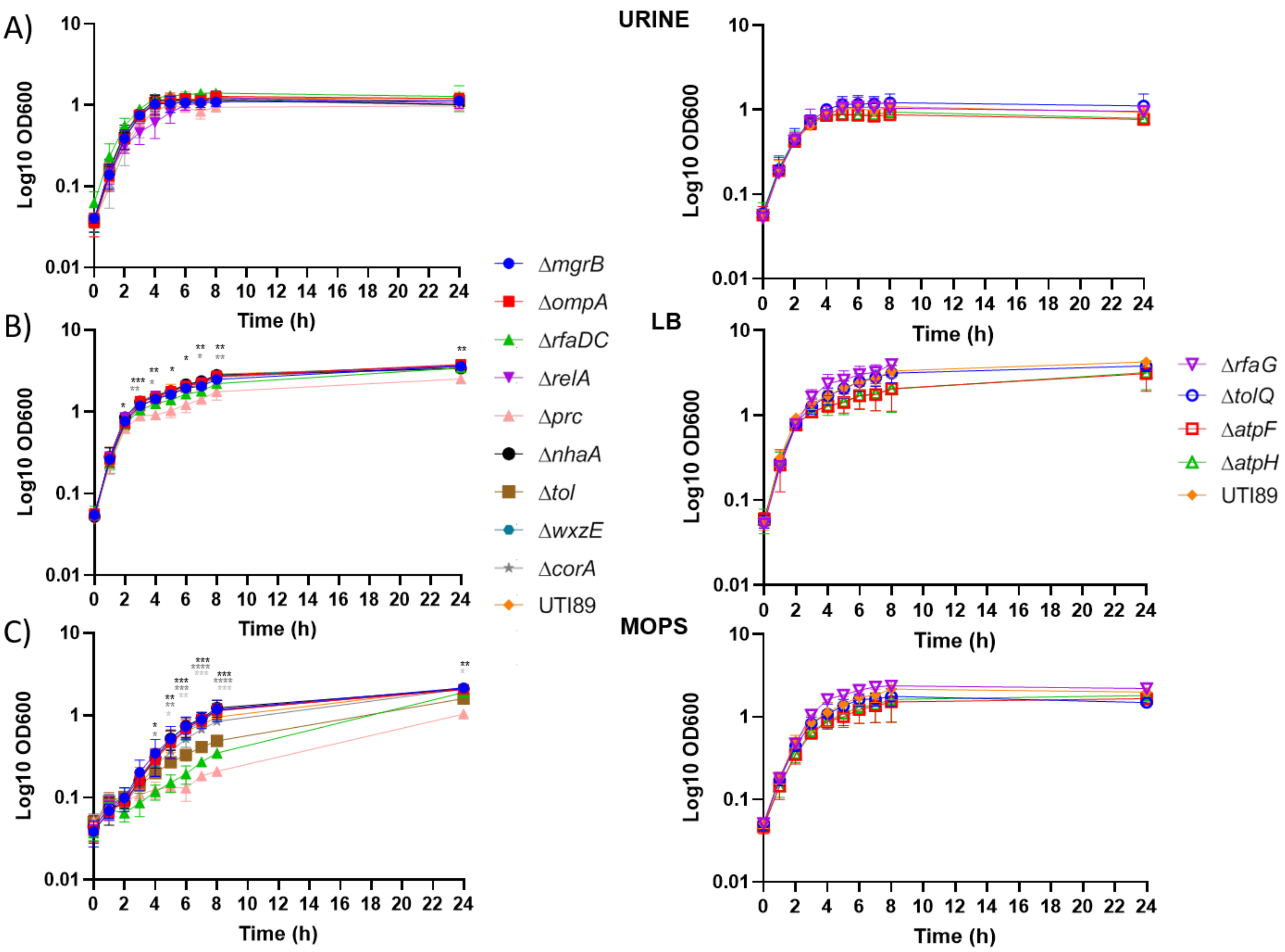

Fig S1. Growth curves obtained for *E. coli* UTI89 and its mutant derivatives in urine (A), LB (B) and EZ-MOPS (C). The data shown are means ± standard deviations of at least three biological replicates. Statistical significance (****p < 0.0001; ***p < 0.001; **p < 0.01; *p < 0.05) was determined by one-sample t-test or ANOVA and Sidak's post-test. Black and dark grey asterisks show significant differences between *E. coli* UTI89 and the mutants Δ*prc* and Δ*rfaDC* in LB and EZ-MOPS. Light grey asterisks show significant differences between *E. coli* UTI89 and the mutant Δ*tol* in EZ-MOPS

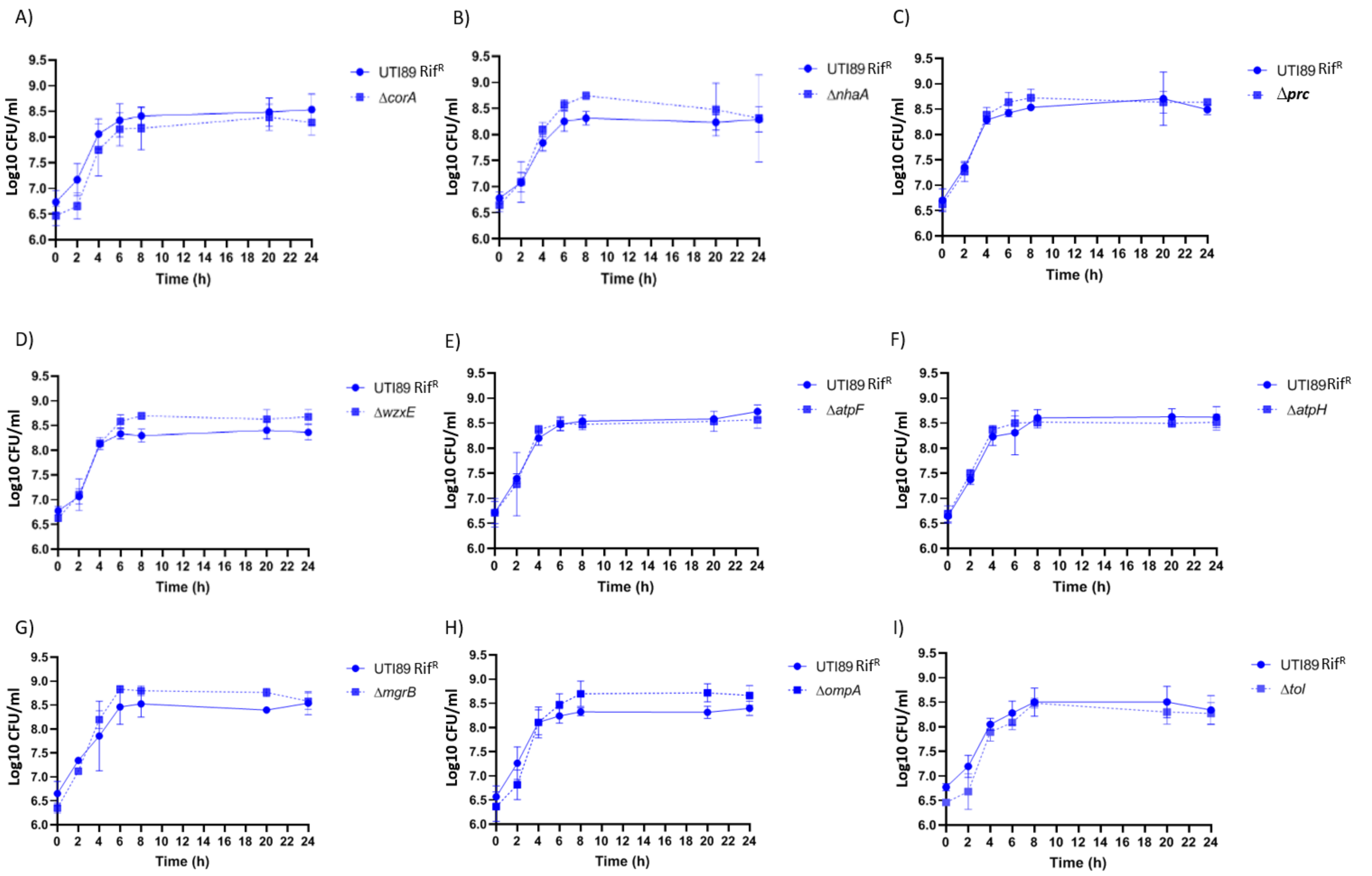

Fig S2. Competition assays of the E. coli UTI89 rifampicin resistant strain (UTI89 RifR) and the mutants ΔcorA (A), ΔnhaA (B), Δprc (C), ΔwzxE (D), ΔatpF (E), ΔatpH (F), ΔmgrB (G), ΔompA (H), Δtol (I), in human urine. The data shown are means ± standard deviations of at least three biological replicates. No statistical significance was observed at any time point between each mutant and UTI89 Rif R**.**

Table S1. UTI89 output libraries and samples obtained in each mouse experiment.

**Output library**	**Sample**	**Cfu/sample**	**Experiment**
UTI89_U	Mouse U	3.5×10^4	N= 11 mice
**UTI89_V**	Mouse V	1.2×10^5	6 h infection
**UTI89_W**	Mouse W	1.0×10^6	5% Glucose 16 h prior to infection
UTI89_X	4 mice (4 bladders)	3.3×10^5	
UTI89_Y	4 mice (4 bladders)	6.9×10^4	
**UTI89_Z**	Mouse Z. One ml of each of the 5 samples (U to Y) (all 11 mice)	8.1×10^6	

Libraries analyzed by TraDIS are highlighted in bold (see Table 2).

Table S2. Primers used for TraDIS.

Primers[1]	Sequence (5'-3')	Reference
SplA5-Top	G*AGATCGGTCTCGGCATTCCTGCTGAACCGCTCTTCCGATC*T	[15]
SplA5-Bottom	/5PHOS/G*ATCGGAAGAGCGGTTCAGCAGGTTTTTTTTTTCAAAAAAA*A	[15]
**Primers[2]**	**Sequence (5'-3')**	
SplAP5.1	C*AAGCAGAAGACGGCATACGAGATAACGTGATGAGATCGGTCTCGGCATTC*C	[15]
SplAP5.2	C*AAGCAGAAGACGGCATACGAGATAAACATCGGAGATCGGTCTCGGCATTC*C	[15]
SplAP5.3	C*AAGCAGAAGACGGCATACGAGATATGCCTAAGAGATCGGTCTCGGCATTC*C	[15]
SplAP5.4	C*AAGCAGAAGACGGCATACGAGATAGTGGTCAGAGATCGGTCTCGGCATTC*C	[15]
SplAP5.5	C*AAGCAGAAGACGGCATACGAGATACCACTGTGAGATCGGTCTCGGCATTC*C	[15]
SplAP5.6	C*AAGCAGAAGACGGCATACGAGATACATTGGCGAGATCGGTCTCGGCATTC*C	[15]
SplAP5.7	C*AAGCAGAAGACGGCATACGAGATCAGATCTGGAGATCGGTCTCGGCATTC*C	[15]
SplAP5.8	C*AAGCAGAAGACGGCATACGAGATCATCAAGTGAGATCGGTCTCGGCATTC*C	[15]
SplAP5.9	C*AAGCAGAAGACGGCATACGAGATCGCTGATCGAGATCGGTCTCGGCATTC*C	[15]
SplAP5.10	C*AAGCAGAAGACGGCATACGAGATACAAGCTAGAGATCGGTCTCGGCATTC*C	[15]
SplAP5.11	C*AAGCAGAAGACGGCATACGAGATCTGTAGCCGAGATCGGTCTCGGCATTC*C	[15]
SplAP5.12	C*AAGCAGAAGACGGCATACGAGATAGTACAAGGAGATCGGTCTCGGCATTC*C	[15]
SplAP5.13	C*AAGCAGAAGACGGCATACGAGATAACAACCAGAGATCGGTCTCGGCATTC*C	[15]
Tn-PCR-final1	AATGATACGGCGACCACCGAGATCTACACCTGATCTAGAGTCGACCTGCAGGCA TGCAAGCTTCAG	[15]
**Primer[3]**	**Sequence (5'-3')**	[15]
qPCR2.1 (P5)	AATGATACGGCGACCACCGAG	[15]
qPCR 2.2 (P7)	CAAGCAGAAGACGGCATACGA	[15]
Tn-seq-primer[5]	AGGCATGCAAGCTTCAGGGTTGAGATGTGTA	[70]
**Primer[4]**	**Sequence (5'-3')**	
iPCRtagSeq	AAGAGCGGTTCAGCAGGAATGCCGAGACCGATCTC	[15]
Illumina Read 1	CGGTCTCGGCATTCCTGCTGAACCGCTCTTCCGATCT	[15]
Tn-seq-primer[5]	AGGCATGCAAGCTTCAGGGTTGAGATGTGTA	[72]

[1]Primers used for adapter ligation

[2]Indexed adapter-specific primers (SpIA5.x) and transposon-specific primer used for PCR enrichment

[3]Primers used for qPCR TraDIS libraries quantification

[4]Primers used for MiSeq Illumina Sequencing

[5]This primer yields specific sequencing of the Tn-insertion sites on the Illumina MiSeq platform while allowing for multiple sample indexing. This primer contained (5′-3′) the P5 sequence (33 bp) to bind to MiSeq flowcells, and the Tn-specific sequence (33 bp) designed to amplify the last 12 bp of the transposon and its adjacent genomic sequence

* Indicates a phosphorothioate group

Table S3. Primers used for the construction and verification of mutants.

Gene/region	Primer[1]	Sequence (5'-3')
corA	corA-KO-F	TTCTGTGTGCCCACCGAACTGTCCGATATTTTAAGCATTGGGAGTCCCGGTCATGCTGAGCGCATTTCAAGTGTAGGCTGGAGCTGCTTCG
	corA-KO-R	ACAATTTTCAGCAATAAGCCGGATAAAGAACAATCTTTACCCGGCATTTATATTACAACCAGTTCTTCCGCATATGAATATCCTCCTTAGTTCC
UTI89_C1262	UTI89_C1262-KO-F	AGGCTGCGTGATAGAATACGCCGCCTTGAAGTTCAATGTCGTGAGTGATCCAATGTCTGAAACCGCAAAAGTGTAGGCTGGAGCTGCTTCG
	UTI89_C1262-KO-R	TAGTAATTCTTTGCCACGTTCACTGCTTCCTTGTTTTAAGTAAAGATAATAATCAGACCGGCAGCGGCAGCATATGAATATCCTCCTTAGTTCC
ybeY	ybeY-KO-F	AACGTAAAGCGGCGCTGGCAGCAGAACGCAAGCGCGAAGAACAGGAACAAAAATGAGTCAGGTGATCCTCGTGTAGGCTGGAGCTGCTTCG
	ybeY-KO-R	CCGTCGTTAATCACCAACGGCGGGGACGTCTGCCAGTCAAATGCCTGGCAAATTATTCTTTCTCGGCAATCATATGAATATCCTCCTTAGTTCC
recB	recB-KO-F	TGGGAGAACGTCAGCGCGTTGCAGCAAACAATGCCCCTGATGAGTGAAAAGAATGAGTGATGTCGCCGAGGTGTAGGCTGGAGCTGCTTCG
	recB-KO-R	GGGCGTAGCTGTTTGTGCTCCACAGCTTCCAGTAATTGCTTTTGCAATTTCATTACGCCTCCTCCAGGGTCATATGAATATCCTCCTTAGTTCC
wzxE	wzxE-KO-F	CCCGTCAATCAGCGTACGGTAATTGCGACCTTGTTGAACTACTTCTCCTGATATGTCGTTGGCAAAAGCGGTGTAGGCTGGAGCTGCTTCG
	wzxE-KO-R	ACGGTTCGGTTATGGTGAGGGATATCCGATCCCAGTACGTGAATCAGTACAGTCATGCCCACCTACGCCACATATGAATATCCTCCTTAGTTCC
nhaA	nhaA-KO-F	CGTGCGGGGTAAAATCGTGAAAACGATCTATTCACCTGAAGAGAAATAAAAAGTGAAACATCTGCATCGAGTGTAGGCTGGAGCTGCTTCG
	nhaA-KO-R	CCTGATAACAATGAAAAGGGAGCCGTTTATGGCTCCCCAGTACATCGTCCTGTCAAACTGATGGACGTAACATATGAATATCCTCCTTAGTTCC
prc	prc-KO-F	TTGATTGTGCGCGCAGAACACCTGGTGTTCTGAAACGGAGGCCGGGCCAGGCATGAACATGTTTTTTAGGGTGTAGGCTGGAGCTGCTTCG
	prc-KO-R	GCATCTTGCCGCTGTTAAAAAATCAGGCACAATTTCTTGTGCCTGATTGATATTACTTGACGGGAGCGGGCATATGAATATCCTCCTTAGTTCC
tol	tolQ-KO-F	TCAAAATGAAGCCTCGTGCGCTTCCCAAGTCTATTGTCGCGGAGTTTAAGCAGTGACTGACATGAATATCGTGTAGGCTGGAGCTGCTTCG
	tolB-KO-R	CACTTTGTTCAGTTGCATTTCAATGATTCCTTTACTATTCAATTAATTATTATCACAGATACGGCGACCACATATGAATATCCTCCTTAGTTCC
tolQ	tolQ-KO-F	TCAAAATGAAGCCTCGTGCGCTTCCCAAGTCTATTGTCGCGGAGTTTAAGCAGTGACTGACATGAATATCGTGTAGGCTGGAGCTGCTTCG
	tolB-KO-R	CACTTTGTTCAGTTGCATTTCAATGATTCCTTTACTATTCAATTAATTATTATCACAGATACGGCGACCACATATGAATATCCTCCTTAGTTCC
relA	relA-KO-F	AATAGTTACGATTTGCCGATTTCGGCAGGTCTGGTCCCTAAAGGAGAGGACGATGGTTGCGGTAAGAAGTGTGTAGGCTGGAGCTGCTTCG
	relA-KO-R	ATGTAGATACAGTATATATCAATCTACATTGTAGATACGAGCAAGTTTCGGCCTAACTCCCGTGCAACCGCATATGAATATCCTCCTTAGTTCC
glnA	glnA-KO-F	ATTGCAGATTTCGTTACCACGACGACAATGACCAATCCAGGAGAGTTAAAGTATGTCCGCTGAACACGTAGTGTAGGCTGGAGCTGCTTCG
	glnA-KO-R	CCACGGCAACTAAATCCCGGCGTTGTTGCGCCGGGTAGTACAACTTCAACTATTAGACGCTGTAGTACAGCATATGAATATCCTCCTTAGTTCC
ompA	ompA-KO-F	ATCTCGTTGGAGATATTCATGGCGTATTTTGGATGATAACGAGGCGCAAAAAATGAAAAAGACAGCTATCGTGTAGGCTGGAGCTGCTTCG
	ompA-KO-R	AGGCGAAAAAAAAACCCCGCAGCTGCGGGGTTTTTCTACCAGACGAGAACTTAAGCCTGCGGCTGAGTTACATATGAATATCCTCCTTAGTTCC
mgrB	mgrB-KO-F	TAACTAACGCATGCTAGTTTAATGACATAAGGTAGGTGAAACGGAGATTGGAGTGAAAAAGTTTCGATGGGTGTAGGCTGGAGCTGCTTCG
	mgrB-KO-R	GCTATTCTACCACTGCTGGAGAGGAAGAAAATCTAGTGCTGAAAAAATGATATCACCACGGGATAAACTGGTCATATGAATATCCTCCTTAGTTCC
eda	eda-KO-F	CTGTATCACTTTTTAAGACGACAAATTTGTAATCAGGCGAGAGAAAACTCTGATGAAAAACTGGAAAACAGTGTAGGCTGGAGCTGCTTCG

	eda-KO-R	CGGCCAAAAAACGCTACAAAAATGCCCGATCCTCGATCGGGCATTTTGACTTTTACAGCTTAGCGCCTTCCATATGAATATCCTCCTTAGTTCC
rfaDC	rfaD-KO-F	AAGTATTCGTGTCTGAGATTGTCTCTGACTCCATAATTCGAAGGTTACAGTTATGATCATCGTTACCGGCGTGTAGGCTGGAGCTGCTTCG
	rfaC-KO-R	ATCAGCAAGGAAGCTGATTTTATTTCAAAATTATCTATTAAAGAGTTGTAATTTAAATCATGGCAGCTTTCATATGAATATCCTCCTTAGTTCC
rfaG	rfaG-KO-F	TCATTGCTGCTGTCGATAAATTACTGCCCTCCTCCACGACAGGTACGTCGTTATGATCGTTGCTTTTTGTGTGTAGGCTGGAGCTGCTTCG
	rfaG-KO-R	TTCCTCAAAAGGATCTTTACCGCGCCATAACGTGGCAAACGGCTCTTTAAGTTCAACCATCCAGACCACCCATATGAATATCCTCCTTAGTTCC
tusA	tusA-KO-F	ACGGACTCACAGGGCGTAAACTAGCGCCGTTTTTTTATGTGATGAGAAGAAAATGACCGATCTCTTTTCCGTGTAGGCTGGAGCTGCTTCG
	tusA-KO-R	GGAGAGGGTTAGGGTGAGGGCAAAAGGCGGCATCGAAGCCAATCTTCCCCCATCAACCGCCTTTACGAATCATATGAATATCCTCCTTAGTTCC
atpH	atpH-KO-F	ACAGCGACATCGTGGATAAACTTGTCGCTGAACTGTAAGGAGGGAGGGGCTGATGTCTGAATTTATTACGGTGTAGGCTGGAGCTGCTTC
	atpH-KO-R	GCTTGATCAGTTCGCTGATTTCGGTGGAATTCAGTTGCATGCTCCAGTCCCCTTAAGACTGCAAGACGTCCATATGAATATCCTCCTTA
atpF	atpF-KO-F	CTTTTATTTAAAGAGCAATATCAGAACGTTAACTAAATAGAGGCATTGTGCTGTGAATCTTAACGCAACAGTGTAGGCTGGAGCTGCTTC
	atpF-KO-R	TTGGCGTAGGGGCGAGCTACCGTAATAAATTCAGACATCAGCCCCTCCCTCCTTACAGTTCAGCGACAAGCATATGAATATCCTCCTTA
ftsE	ftsE-KO-F	CGTTTAAGGCGGACGACTTTATAGAGGCACTTTTTGCCCGAGAGGATTAACAATGATTCGCTTTGAACATGTGTAGGCTGGAGCTGCTTC
	ftsE-KO-R	GAAGCGATCCAGACGCCCGCCAAACTGCCGAATATGATTGATTGCATCGCGCTTATTCATGGCCCACGCCCATATGAATATCCTCCTTA
ypdE	ypdE-KO-F	CAGAGGTGCTCTACGCCATGCCGAAAACAGTGTTGCTCACGGGAGAGGCATAATGGATTTATCGCTATTAGTGTAGGCTGGAGCTGCTTC
	ypdE-KO-R	AAGGACAGAGAAATTGAATCGTTAACATAACCGCTCCGGGTTAATCAGGAGATCATCTGAAATCCGTCAGCATATGAATATCCTCCTTA
himD	himD-KO-F	TCAATGCAGCAACAGCAGCCGCTTAATTTGCCTTTAAGGAACCGGAGGAATCATGACCAAGTCAGAATTGGTGTAGGCTGGAGCTGCTTC
	himD-KO-R	TGAAAAAAGCACCCGACAGGTGCTTTTCTCGCGTTCAAGTTTGAGTAAAAACTTAACCGTAAATATTGGCCATATGAATATCCTCCTTA
cutA	cutA-KO-F	CGTTGTATAGTGACCTCTCTCTTGCGGTTCCATCTGTTCTTGCGAGGTGTTTATGCTTGATGAAAAAAGTGTGTAGGCTGGAGCTGCTTC
	cutA-KO-R	CCCGGCGCGTCGAATAATCCGGCAAAAACGGAAGTGCTGCAAAGTAGCAGGATCAGCGTAAAGATGCGTTCATATGAATATCCTCCTTA
phnO	phnO-KO-F	CGCCAGTCGGTCGACACGCTGCTGACGCTGATTCATCAGAAGGAGAAACACCATGCCTGCTTGTGAACTTGTGTAGGCTGGAGCTGCTTC
	phnO-KO-R	CGGAACGCCCTGTGCGCCGCCGGTGCCGGTGAGCGTGAGGGTCAGGCTCATGTTACAGCGCCTTGGTGAAGCCATATGAATATCCTCCTTA
tam	tam-KO-F	AATTTATCAGTTTTATCTACAATTGGGGTAACGCGCTGACGGGAGTAAAAAAATGTCTGACTGGAACCCCGTGTAGGCTGGAGCTGCTTC
	tam-KO-R	AGAAATTCCAGCGAAAATTCTTCCCGATCGCCATTACCAGCTGACGTGATAATTACTCCGTACGGCGGGCCATATGAATATCCTCCTTA
sufA	sufA-KO-F	ATTATCACTAACATGCTGTTATACGCTGAAAGCGATGAAGTGAGGTAAATCGATGGACATGCATTCAGGAGTGTAGGCTGGAGCTGCTTC
	sufA-KO-R	GTCCAGGTTTTGACATCGTCAGTTGCTTCAGTATTACGAGACATAGTACCGCCTATACCCCAAAGCTTTCCATATGAATATCCTCCTTA
**Gene/Region**	**Primer[2]**	**Sequence (5'-3')**
corA	corA-check-F	ATAGCCTTAGCGGTTGTCAG
	corA-check-R	GATAGAGCGAGTGTTCAACC
UTI89_C1262	UTI89_C1262-check-F	ATCCGCTGGAGATGATTGGC
	UTI89_C1262-check-R	CTGACAAATACCGGCAAGGG

ybeY	ybeY-check-F	TTAACGCCTATGAAGCCTGG
	ybeY-check-R	GCGTCGTATGGGTTCTCATG
recB	recB-check-F	ACGGGAAAGCCGAATATGTG
	recB-check-R	GACGGCAGGATGTTCATCTC
wzxE	wzxE-check-F	GCCTGCCGTTGTTCTACAAC
	wzxE-check-R	CCAGCGCGTCGTTGAAAAAC
nhaA	nhaA-check-F	TCTCGCTGATGGCGCAATTC
	nhaA-check-R	CCAACCACAGAACCTTCTTTG
prc	pcr-check-F	AATCACCAAAGACGGCGTCC
	pcr-check-R	CATCGTATGGGTTACGCGAC
tol	tol-check-F	TCTGGTTGTTTGCGTTGACC
	tol-check-R	AGGCAGAGCAATCATCAGCC
tolQ	tolQ-check-F	TTTTATGAAAGAGCACGCACAGA
	tolQ-check-R	CCAGACACTTCAACAATCACTGG
relA	relA-check-F	GATGGTACTTTTCTCGCGCG
	relA-check-R	AACCCTTTCCTCAAACCGCG
glnA	glnA-check-F	TGATAACGCCTTTTAGGGGC
	glnA-check-R	ATGCAGAGATGGGCTGAAAG
ompA	ompA-check-F	TTTTTCATATGCCTGACGGAGTT
	ompA-check-R	CGATCGATCTGGAATTTATCGTC
mgrB	mgrB-check-F	CATTCCGAAAAAGCACGAATATC
	mgrB-check-R	AAGAAGAACCACCACCGATACAA
eda	eda-check-F	ACATCATTCGTGTGAATGGACAG
	eda-check-R	TGATAATGTGCAGAATGCCGTAG
rfaDC	rfaD-check-F	CAAAAGTTAATGACGTGGCTTCC
	rfaC-check-R	GTTATCAACGCGTGGTATTCCTG
rfaG	rfaG-check-F	GTTTGGTGCGACAGACCATATTT
	rfaG-check-R	TGCCACCCCATACATAGTATCAA
tusA	tusA-check-F	ACGATAACCAGAACAACGCCTTA
	tusA-check-R	GAGGCTATCAGTCTACGGTCCAA
atpH	atpH-check-F	AACGAGCACATAAGGACCTTGAC
	atpH-check-R	GGAGATCATTTCACCCTGCATAC
atpF	atpF-check-F	CTGGAGACTGTCATGGAAAACCT
	atpF-check-R	AAGACGACCATTTTCAGCCATTA
ftsE	ftsE-check-F	ATGAAGAAACTCGACGTTGAAGC
	ftsE-check-R	ATTTGCGGTGACGGATAATACTG
ypdE	ypdE-check-F	ACCCGCTGTTTAACGTCTATCAA

	ypdE-check-R	TTCAGGCTGCAACTGTCATTAAA
himD	himD-check-F	TCGGGATTTTTATTCCGAAGTTT
	himD-check-R	GAGAGTCAGAAACGCAAGGGTTA
cutA	cutA-check-F	ACCCGTATCGGTAAATTCGTCTT
	cutA-check-R	ACAGCCCTGGTAGGTGACAGTTA
phnO	phnO-check-F	GTTTGTATTACGGTGTCGGCATT
	phnO-check-R	GTGATCCATATGGTAGTGCGTCA
tam	tam-check-F	CATGTCATACGTCCACAGCAAAT
	tam-check-R	ATCTGCATTACATGACGCCTTTT
sufA	sufA-check-F	AAGAGCTAAGCCACTGTGAGTGC
	sufA-check-R	GTGATAGGCGTTGAGACGAAACT

[1]Primers used for construction of mutants

[2]Primers used for verification of mutants
